# Secretome of senescent hepatic stellate cells favors malignant transformation from nonalcoholic steatohepatitis-fibrotic progression to hepatocellular carcinoma

**DOI:** 10.7150/thno.85369

**Published:** 2023-08-06

**Authors:** Yuan Zhou, Li Zhang, Yue Ma, Li Xie, Yong-yu Yang, Cheng Jin, Hui Chen, Ying Zhou, Guang-qi Song, Jia Ding, Jian Wu

**Affiliations:** 1 Department of Medical Microbiology & Parasitology, MOE/NHC/CAMS Key Laboratory of Medical Molecular Virology, School of Basic Medical Sciences, Fudan University, Shanghai 200032, China.; 2 Department of Gastroenterology, Shanghai Jing'an District Central Hospital, Fudan University, Shanghai 200040, China.; 3 Joint Laboratory of Biomaterials and Translational Medicine, Puheng Technology Co., Ltd, Suzhou 215163, China.; 4 Department of Gastroenterology & Hepatology, Zhongshan Hospital, Fudan University, Shanghai 200032, China.; 5 Shanghai Institute of Liver Diseases, Fudan University Shanghai Medical College, Shanghai 200032, China.

**Keywords:** Nonalcoholic steatohepatitis, Hepatocellular carcinoma, Hepatic stellate cell, Senescence, Senescence-associated secretary phenotype

## Abstract

**Background:** Hepatic fibrosis is a premalignant lesion, and how injured hepatocytes transform into malignancy in a fibrotic microenvironment is poorly understood. Senescence is one of major fates of activated hepatic stellate cells (HSCs). Paucity of literature is available regarding the influence of senescent HSCs on behavior of steatotic hepatocytes.

**Methods:** Senescent HSCs were identified in a murine model of nonalcoholic steatohepatitis (NASH)-fibrosis-hepatocellular carcinoma (HCC) and human NASH-HCC specimens. Secretome of senescent HSCs was analyzed by label-free mass-spectrum (NanoRPLC-MS/MS) and verified quantitatively.

**Results:** Senescent HSCs were increased along with the progression from nonalcoholic fatty liver (NAFL), NASH to NASH-fibrosis, and reached a peak at the stage of advanced fibrosis and then decreased when hepatocellular dysplasia or HCC was developed. Critical components affecting proliferation, epithelial-mesenchymal transition (EMT) or migration were identified from secretome of senescent HSCs, and may activate morphogenic hedgehog or oncogenic Wnt signaling pathways to accelerate malignant transformation of steatotic or dysplastic hepatocytes. Primary hepatocytes stimulated with conditioned medium from senescent HSCs, in co-culture or co-cultured in 3D spheroids with senescent HSCs exhibited an enhanced proliferating or EMT profile.

**Conclusion:** Senescent HSCs secreted a characterized protein profile favoring malignant transformation of steatotic or dysplastic hepatocytes through activating morphogenic hedgehog or oncogenic Wnt signaling pathways in the progression from NASH to malignancy.

## Introduction

As the 4th most common malignancy, liver cancer is the second cause of death among all cancers in China. Its incidence will be doubled in the next decade in Europe and the US, and nonalcoholic fatty liver disease (NAFLD) will become the major base illness contributing to the increased incidence of liver cancer in the US in the next two decades 1. Various modes of chronic liver injury accompany with progression of hepatic fibrosis, which is considered to be a premalignant lesion of liver cancer 2. It has been documented that hepatocellular carcinoma (HCC) may occur in nonalcoholic steatohepatitis (NASH) with fibrotic progression even without cirrhosis 3. Certainly, HCC incidence increases dramatically when cirrhosis is developed. Questions remain how hepatic fibrosis triggers the initiation or acceleration of malignant transformation of damaged hepatocytes with steatotic or ballooned degeneration in an inflamed and fibrotic microenvironment?

Growing evidence has suggested that activated hepatic stellate cells (HSCs) may play a pivotal role in releasing cytokines that exhibit proliferative, angiogenic or proinflammatory actions, such as platelet-derived growth factor (PDGF), endothelin-1, CCR2/5, IL-6, leptin, activin, osteopontin (OPN), transforming growth factor-α (TGF-α), fibroblast growth factor (FGF), etc. to hepatocytes, endothelial cells or function as attractants to inflammatory cells 4-6. As two major fates of HSC activation, both apoptosis and senescence of activated HSCs are therapeutic goals in alleviating hepatic fibrosis 7, 8. In fact, enhanced apoptosis or accelerated senescence of HSCs appeared to be effective approaches in minimizing hepatic fibrosis in rodent models 9, 10. However, paucity of literature is available regarding possible influence of senescent HSCs on hepatocellular behaviors, especially in fate changes, such as malignant transformation.

As a mesenchymal cell type, HSCs undergo a phenotypic transformation from a quiescent state to myofibroblast-like cells in response to a variety of chronic liver injury, become proliferative, contractive and migratable, produce an excessive amount of extracellular matrix compounds, and release various factors, such as cytokines or intermediate substances, which act on themselves or neighboring hepatocytes, sinusoidal endothelial cells (LSEC) or other cell types 11, 12. Hence, HSCs are considered as the effector cell type in the initiation and progression of hepatic fibrosis 5. Although it is possible to induce a reversal of activated HSCs to a quiescent state in experimental settings, apoptosis and/or senescence are two major natural fates in the regression of hepatic fibrosis after successful antiviral treatment or resolution of NASH 13. From mesenchymal stem cells (MSCs), it is learnt that senescent MSCs may change their secretome to a specific series of inflammatory cytokines, chemokines, growth factors, and matrix-remodeling factors known as senescence-associated secretary phenotype (SASP) to affect the local environment and contribute to chronic inflammation 14, which may occur to HSCs in the transition from NASH to fibrosis, further to HCC with gut dysbiosis and toxicity of deoxycholic acid (DCA) 15. Although studies indicated that SASP was possible factors that might favor the initiation or acceleration of malignant transformation of injured hepatocytes in an inflamed and fibrotic microenvironment, there is no systemic investigation of proteomic changes of senescent HSCs and define critical factors in supporting the speculation.

In the present study, senescent HSCs were observed in a well-established murine model of NASH-fibrosis-HCC and human NASH-HCC specimens, and altered secretome of senescent HSCs induced by two different approaches was quantitatively analyzed by label-free mass-spectrum (NanoRPLC-MS/MS); and critical factors released from senescent HSCs were verified by ELISA. Moreover, the conditioned medium from senescent HSCs, co-culture of hepatocytes or co-cultured in 3D spheroids with senescent HSCs further demonstrated a proliferating or epithelial-mesenchyamal transitional (EMT) status of primary hepatocytes under the influence of senescent HSCs. These findings underscore favorable action from critical factors identified in the secretome of HSCs for the initiation and acceleration of malignant transformation, and imply key molecular targets in the prevention of fibrotic progression to hepatocellular malignancy.

## Materials and Methods

### Patient tissue samples

The human NASH-HCC specimens were obtained during routine surgical procedures at Huashan Hospital of Fudan University (Shanghai, China). The use of the liver cancer specimens was approved by the Ethic Committee of Fudan University School of Basic Medical Sciences (2019-C006) and informed consent was given in writing by all subjects. The histopathological diagnosis of liver cancer was based on the World Health Organization criteria by hematoxylin and eosin (H&E) staining. Steatosis without cirrhosis in peri-carcinoma tissues was confirmed after exclusion of HBV, HCV, alcoholic, and any other genetic or non-genetic liver diseases based on the patient's history and laboratory test results.

### Mouse models of NASH-HCC

Male C57BL/6J mice aged 6-8 weeks were purchased from Nanjing Biomedical Research Institute of Nanjing University (Nanjing, China). Mice were randomly divided into 2 diet feeding groups: HFCD-HF/G group (high-fat/calorie diet plus high fructose/glucose in drinking water) and control diet group. Each group was further divided into 5 subgroups based on the diet feeding duration: 2, 5, 9, 12 and 14 months, representing pathologic verification of NAFL, NASH, NASH with fibrosis, advanced fibrosis with dysplasia and HCC in these mice. Mice in HFCD-HF/G group were fed a high fat calorie diet (D12492i; Research Diets, New Brunswick, NJ, USA) plus drinking water with a final concentration of 42 g/L fructose/glucose containing 55% high fructose (23.1 g/L, F3510; Sigma, St Louis, MO, USA) and 45% glucose (18.9 g/L, G8270; Sigma, St Louis, MO, USA) 16. Mice in control group were fed a chow pellet diet containing 12% kCal from fat (1010010; Xietong Organism, Nanjing, China) with regular drinking water. Animal experiment procedures (#2018-0302-072) were approved by the Ethic Committee of Fudan University School of Basic Medical Sciences and all procedures were performed following the NIH Guidelines of Experimental Animal Handling and Use.

### *In vitro* induction of senescence in HSCs

Rat immortalized HSC BSCC-10 cells were used in the induction of senescence. BSCC-10 cells were obtained from Prof. Hidekazu Tsukamoto, Keck School of Medicine of University of Southern California, Los Angeles, CA, USA 17; are partially-activated HSCs and exhibit spontaneous immortalization without transfection of human telomerase reverse transcriptase (hTERT) genes 18, which is different from other immortalized human HSC lines, such as LX2 or hTERT-HSCs 19, 20. BSCC-10 cells were cultured in DMEM with 10% fetal bovine serum (FBS, Gibco Life Technologies, Grand Island, NY, USA) and 1% penicillin-streptomycin at 37 °C with 5% CO_2_ in air. Two approaches were employed to induce senescence of HSCs: treatment with a DNA damage agent, etoposide (ETP) or serial passages. ETP (HY-13629; MedChemExpress, Monmouth Junction, NJ, USA) was first dissolved in dimethyl sulfoxide (DMSO) and added to the medium at the final concentration of 100 μM. BSCC-10 cells were seeded at 1 × 10^5^ in 6-well plates for 24 h and then exposed to ETP at 100 μM for 48 h. For serial passages, BSCC-10 cells were subcultured using 0.25% trypsin/EDTA (Gibco Life Technologies, Grand Island, NY, USA) when they reached 80% confluence, and finally passaged up to the 30^th^ generation when senescence-associated β-galactosidase (SA-β-Gal) staining verified that sufficient proportion of cells was β-Gal-positive.

### Treatment with fatty acids

Rat immortalized BSCC-10 HSCs were exposed to a combined overload of palmitic acid (PA) with oleic acid (OA). BSCC-10 cells were seeded at 1 × 10^5^ in 6-well plates for 24 h and treated with PA and OA for 48 h. PA and OA were first dissolved in methanol and added to the medium at the final concentration of 200 μM and 400 μM.

### Isolation and culture of rat primary HSCs

Primary HSCs were isolated as reported previously 21 from male Sprague-Dawley rats weighing at 500 g. Briefly, rats were perfused with Ca^2+^, Mg^2+^-free solution, pronase E and collagenase type IV solution. Liver was digested with DNase solution and HSCs were separated by gradient centrifugation. Primary HSCs were seeded at 12-well plates after isolation and cultured in M199 medium (Hyclone, Marlborough, MA, USA) containing 20% FBS and 1% penicillin-streptomycin at 37 °C with 5% CO_2_ in air, and FBS concentration was changed to 10% after overnight culture. Immunofluorescent staining was performed 4 days after isolation.

### Isolation and culture of primary mouse hepatocytes

Mouse primary hepatocytes were isolated using a two-step collagenase perfusion protocol as reported previously 18 from male C57BL/6J mice aged 8-10 weeks, weighing at 25 g. Briefly, mouse liver was perfused with Hank's buffered saline solution (HBSS) and collagenase buffer. Hepatocytes were seeded at collagen I-precoated 12-well plates or Transwell inserts after isolation and cultured in Williams E medium containing 10% FBS and 1% penicillin-streptomycin at 37 °C in atmosphere with 5% CO_2_. HSC supernatant stimulation and co-culture with HSCs were performed 24 h after isolation.

### Co-culture of hepatocytes with HSC

Mouse primary hepatocytes were seeded at 1 × 10^5^ onto top transwell inserts with a polyethylene terephthalate membrane pore size of 0.4 μm in 12-well plates (Costar 3460; Corning, Corning, NY, USA). Growing or senescent HSCs were seeded at 1 × 10^5^ on the bottom section of the plate. Total RNA was extracted from hepatocytes 48 h after the Transwell culture.

### RNA extraction and qRT-PCR

Total RNA was isolated according to the manufacturer's procedure from growing or senescent HSCs and mouse primary hepatocytes by Trizol reagent (Invitrogen, Carlsbad, CA, USA). RNA was then reversely transcribed into cDNA using a PrimeScript RT reagent kit (RR037A; Takara Bio, Shiga, Japan). Quantitative reserve transcriptase polymerase chain reaction (qRT-PCR) was performed using a Power SYBR green PCR Master Mix (4368708; Applied Biosystems, Bedford, MA, USA). Relative gene expression levels were calculated using 2^-ΔΔCt^ method and normalized by rat or mouse glyceraldehyde-3-phosphate dehydrogenase (GAPDH) or β-actin as house-keeping gene controls 22. Sequences of all primers used in qRT-PCR were listed in **Table S1**.

### SA-β-Gal staining

Senescence-associated β galactosidase (SA-β-Gal) staining was performed using a senescence β-galactosidase (β-Gal) staining kit (C0602; Beyotime, Shanghai, China) according to the manufacturer's procedure. Briefly, frozen liver sections and cells grown on the glass slide were first fixed with fixation solution containing formalin and incubated with SA-β-Gal staining solution at 37 °C overnight. All cells in 5 randomly-selected fields at 200× magnification were used to quantify a positive senescent ratio with Image J software for each section.

### Double staining of SA-β-Gal with α-SMA

Double staining of SA-β-Gal staining with smooth muscle α-actin (α-SMA) was performed according to a protocol reported previously 23. Briefly, SA-β-Gal staining was first performed in frozen liver sections as described above. After incubation with SA-β-Gal staining solution, sections were blocked with 5% bovine serum albumin (BSA; Sigma, St Louis, MO, USA) in PBS for 2 h and incubated with anti-α-SMA antibody at 4 °C overnight. Sections were then treated with 3% H_2_O_2_ at room temperature for 60 min to block endogenous peroxidase activity. The positive signal was developed with DAB color rendering (DAB) horseradish peroxidase color development kit (G1212-200T; Servicebio, Wuhan, China). Five randomly-selected fields at 400× were used to quantify senescent HSCs with Image J software for each section.

### Immunofluorescent staining of liver sections

Frozen liver sections were fixed with 4% paraformaldehyde and incubated with 0.25% Triton X-100 at room temperature for 10 min. For nucleoprotein like Gli-1, mixture of glacial acetic acid and ethanol (volume/volume 1:3) was used to increase the permeability of primary antibodies into nuclear membrane. Sections were blocked with 5% BSA at room temperature for 30 min and incubated with primary antibodies in 1% BSA at 4 °C overnight. On the second day, sections were incubated with secondary antibody at 37 °C for 1 h, and nucleus was counter-stained with 4'6-diamidino-2-phenylindole (DAPI) (C1005; Beyotime, Shanghai, China). Cells in 5 randomly-selected fields at 630× under a confocal microscope (Nikon Corporation, Tokyo, Japan) were used to quantify with Image J software for each section. Commercial sources of all antibodies used in this study are listed in **Table S2**.

### Verification of secreted proteins by ELISA

To determine the release of TGF-β1, connective tissue growth factor (CTGF), PDGF-BB, insulin-like growth factor 1 (IGF1), Wnt10b and sonic hedgehog-N ligand (SHh-N) into medium, medium supernatant from HSC culture was collected, and concentration of released factors listed above was then determined according to the manufacturer's protocol with commercially available ELISA kits. The sources of ELISA kits used in this study were listed in **Table S3**.

### Determination of secretome profiling by NanoRPLC-MS/MS

Growing or senescent BSCC-10 cells were seeded at 1 × 10^5^ in 6-well plates for 24 h and cultured in 1 ml of serum-free DMEM for another 48 h to collect supernatant medium. Total protein at 20 μg was loaded into a gel for electrophoresis after protein quantification and Coomassie staining was performed on the gel. Then the region containing protein was excised, reduced with trichloroethyl phosphate, alkylated with iodoacetamide, and digested by trypsin and desalted using C18 ZipTips (Millipore, Billerica, MA, USA) before liquid chromatography-mass spectrometry (LC-MS/MS). The determination of secretome profiling was conducted by Institutes of Biomedical Sciences of Fudan University (Shanghai, China) 24. Secretome profiling was performed via label-free quantitative proteomics on an UltiMate 3000 RSLCnano LC-MS system (Thermo Scientific, San Jose, CA, USA) connected to an Orbitrap Fusion Lumos mass spectrometer (Thermo Scientific, San Jose, CA, USA) equipped with an online nanoelectrospray ion source. In LC procedure, mobile A and B phases were water with 0.1% formic acid and acetonitrile with 0.1% formic acid. Peptides were separated on the analytical column (Acclaim PepMap C18, 75 μm × 15 cm) with a 120 min gradient and mobile phase B from 2% to 30% in 105 min. The column was re-equilibrated at initial conditions for 15 min with maintainable column flow rate at 300 nL/min. In MS procedure, MS1 was performed at a resolution of 120,000 with mass-to-charge ratio between 350 and 1550 m/z. MS/MS analysis was performed in Orbitrap system with a cycle time of 3 s, a resolution of 15,000 and an intensity threshold of 50,000. Ions were fragmented by higher energy collisional dissociation with a collision energy of 35% and a dynamic exclusion time of 30 s. The data were analyzed based on the UniProt rat database. Bioinformatic analysis was performed using the OmicStudio tools. Protein-protein network was analyzed using the STRING database and drawn by Cytoscape software.

### 3D liver spheroid construction

3D liver spheroids were constructed with activated (BSCC-10 cells for the control group) or senescent (BSCC-10 cells passaging to F30 for the senescent group) HSCs and rat primary cells in 384-well Corning as reported previously 25. Both cell types were assembled with DNA origamis in a ratio of 1:1 (3000 cells per spheroid). The formed spheroids were cultured for 2 weeks.

### Statistical analysis

All data were expressed as mean ± SEM. Stata 10.0 was used for statistical analysis. In comparison of two groups, student *t*-test was used after normal distribution test. If normality or homogeneity of variance is not met, Mann-Whitney U test is used between two groups. One way analysis of variance tests (ANOVA) was used to analyze between more than 2 groups and least significant difference (LSD) test was used to analyze multiple comparisons between two given groups. If normality or homogeneity of variance is not met, Kruskal-Walli's test is used to compare between groups and Hodges-Lehmann test was used between two given groups. All experiments were repeated for three times or with three biologically independent samples. *p* value less than 0.05 was considered statistically significant.

## Results

### Increased senescent myofibroblast-like cells during progression of NASH-HCC

**Figure 1:**
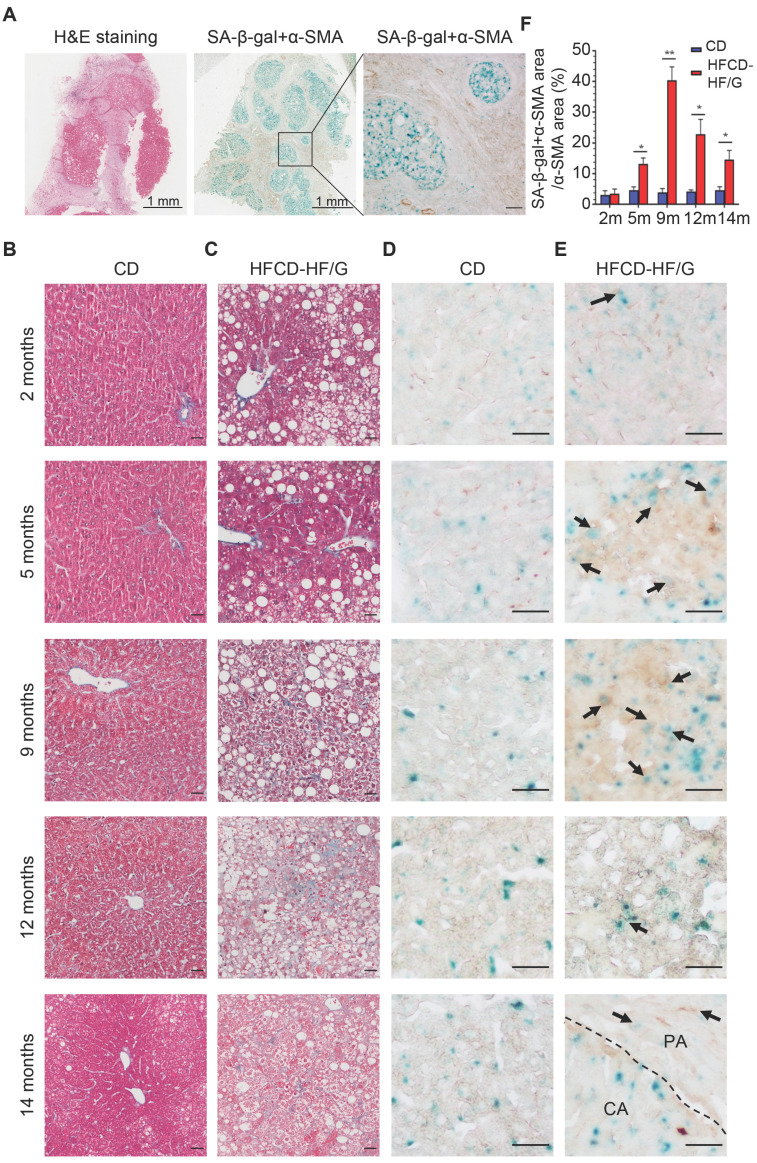
** Increased senescent HSCs during the progression of NASH-HCC. (A)** H&E and double staining of SA-β-Gal with α-SMA in a human NASH-HCC specimen. The first two entire section images were taken at original magnification (20×). Scale bars = 1 mm. The third image was taken at original magnification (200×). Scale bars = 100 μm. **(B)** Representative micrographs of Masson Trichrome staining of liver tissues at 2, 5, 9, 12 and 14 months of the control diet-fed mice and **(C)** HFCD-HF/G diet-fed mice. Images were taken at original magnification (200×). Scale bars = 100 μm. **(D)** Representative micrographs of double staining of SA-β-Gal with α-SMA of liver tissues at 2, 5, 9, 12 and 14 months in the control diet and **(E)** HFCD-HF/G diet-fed mice. The positivity of counter-staining was developed with DAB and SA-β-Gal (in both brown and blue color indicated by arrow symbols). The areas of cancerous (CA) and paracancerous (PA) tissue were indicated by labels and divided by dashed lines of the section from mice fed HFCD-HF/G diet at 14 months. Images were taken at original magnification (400×). Scale bars = 100 μm. **(F)** The semi-quantitative analysis of dynamic changes in senescent HSC number during the progression from NASH to fibrosis, further to HCC in mice. All data were expressed as mean ± SEM. *p < 0.05 and **p < 0.01 compared to control diet-fed mice at corresponding time points.

**Figure 2:**
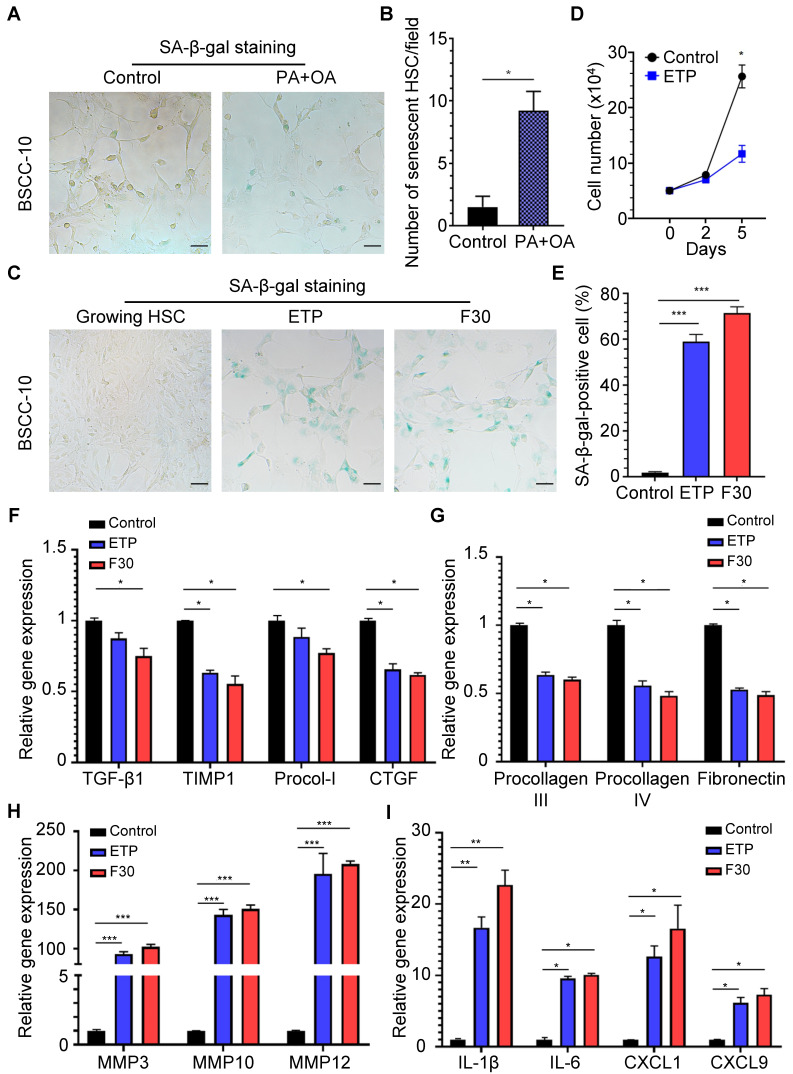
** Effects of rat immortalized HSCs treated with fatty acids and features of senescent HSCs *in vitro*. (A)** Representative micrographs of SA-β-Gal staining of rat BSCC-10 cells treated with palmitic acid (PA) at 200 μM and oleic acid (OA) at 400 μM for 48 h. Images were taken at original magnification (200×). Scale bars = 50 μm. **(B)** Quantitative count of senescent HSCs following treatment with PA and OA. *p < 0.05 compared to control. **(C)** SA-β-Gal staining of two approaches to induce senescent HSCs *in vitro* including etoposide (ETP) treatment and serial passages to the 30^th^ (F30) generation. Images were taken at original magnification (200×). Scale bars = 50 μm. **(D)** The quantitative analysis of cell proliferation in 5 days. **(E)** The percentage of senescent HSCs under ETP treatment or serial passages over time. **(F)** Relative mRNA levels of markers of HSC activation (TGF-β1, TIMP1, procollagen I and CTGF) in senescent HSCs. **(G)** Relative mRNA levels of extracellular matrix (ECM) components (procollagen III, IV and fibronectin) in senescent HSCs. **(H)** Relative mRNA levels of matrix metalloproteinases (MMP3, MMP10 and MMP12) in senescent HSCs. **(I)** Relative mRNA levels of IL-1β, IL-6, CXCL1 and CXCL9 in senescent HSCs. All data were expressed as mean ± SEM. *p < 0.05, **p < 0.01 and ***p < 0.001 compared to controls (growing HSCs with no treatment).

In order to investigate the existence of senescent myofibroblast-like cells in the progression of NASH-HCC, double-staining of SA-β-Gal with α-SMA, markers of senescent cells and activated HSCs, was performed in a human NASH-HCC specimen. Senescent myofibroblast-like cells were mainly observed in the fibrous tissue encasing the malignant cell clusters. At the same time, a large portion of malignant cells in clusters were SA-β-Gal-positive (**Figure 1A**). To investigate the dynamic changes of senescent myofibroblast-like cells during NASH-HCC progression, liver specimens of a mouse model of NASH-fibrosis-HCC collected at 2, 5, 9, 12 and 14 months of HFCD-HF/G feeding were stained in parallels, representing the time points of NAFL, NASH, NASH-fibrosis, advanced fibrosis with dysplasia and appearance of HCC. Masson's trichrome staining documented that hepatic fibrosis was observed at 5 months (**Figure 1C**) and worsened at 9 months in the extension of HFCD-HF/G feeding, advanced fibrosis with dysplasia at 12 months 16, and finally progressed to HCC at 14 months (**Figure 1C**, **Figure S1**). The double-staining of markers of senescent cells including SA-β-Gal (**Figure 1D-E**), p16 and p21 (**Figure S2**) with α-SMA were counterstained on the same sections. Compared to control diet-fed mice, senescent myofibroblast-like cells were significantly increased along with extension of feeding time in HFCD-HF/G-fed mice. Clusters of activated HSCs were observed at both 5 and 9 months of HFCD-HF/G feeding, however there exists difference that senescent cells started to appear at 2 months (**Figure 1E**) and became much more at 5 months in areas surrounded with activated HSCs (**Figure 1E**); whilst number of senescent myofibroblast-like cells reached the highest in these α-SMA-positive areas at 9 months (**Figure 1E**). The positive ratio of senescent HSCs over α-SMA-positive areas continued to increase from 2 to 9 months, reaching a peak of 40.41% at 9 months and decreased at 12 months (22.86%) when precancerous lesions occurred and further decreased at 14 months (14.58%) when finally progressed to HCC (**Figure 1F**). The same pattern was seen over time in the immunofluorescent staining of p16 and p21, both of which were considered as additional markers of senescence (**Figure S2**). The dynamic change of senescent HSCs was highlighted as more HSCs became active in the progression of NASH-HCC and thus led to more senescence after activation. To confirm that the increase of senescent HSCs was associated with the sustained high fat feeding, rat immortalized BSCC-10 cells were treated with palmitic acid (PA) at 200 μM plus oleic acid (OA) at 400 μM for 48 h. The number of senescent cells was significantly increased after the overload of PA plus OA compared to control (p < 0.05; **Figure 2A-B**). Therefore, fatty acid overload in HSCs may enhance HSC senescence *in vitro*.

### Senescence-associated changes in genes and secretory factors from senescent HSCs

To investigate the role of senescent HSCs on progression of NASH-HCC, two approaches were employed to induce senescent HSCs *in vitro*: treatment with DNA damage agent, etoposide (ETP) and serial passages of HSC culture up to 30 generations. ETP-treated HSCs exhibited a high ratio of SA-β-Gal positivity and cellular growth arrest (**Figure 2C-D**). After 30th passage, HSCs (F30) displayed a slightly higher SA-β-Gal positivity than those treated with ETP (**Figure 2C-E**, p>0.05).

From a genetic perspective, mRNA levels of markers for HSC activation, such as TGF-β1, TIMP-1, procollagen type I (Procol-I) and CTGF were decreased in both ETP-treated and F30-HSCs compared to control cells (**Figure 2F**). Functionally, mRNA levels of extracellular matrix (ECM) components, including procollagen III, procollagen IV and fibronectin were decreased (**Figure 2G**); however, gene expression of matrix metalloproteinase (MMP) genes capable of degrading ECM, including MMP3, MMP10 and MMP12, was remarkably increased with rising levels to be more than one hundred-fold (**Figure 2H**). It was reported that senescence-associated changes in gene expression were characterized and gene expression including pro-tumorigenic protein was elevated in senescent HSCs 26. In consistency with these studies, ETP-treated and F30-HSCs exhibited higher levels of pro-inflammatory gene expression, such as IL-1β, IL-6, CXCL1 and CXCL9, as well as MMPs (for tumor metastasis) than controls (**Figure 2I**). The changes of gene expression profile in senescent HSCs confirmed that senescent HSCs were successfully induced, and the increased gene expression of several pro-inflammatory proteins, such as IL-1β, IL-6, CXCL1 and CXCL9, might be involved in the progression of NASH-fibrosis-HCC.

**Figure 3:**
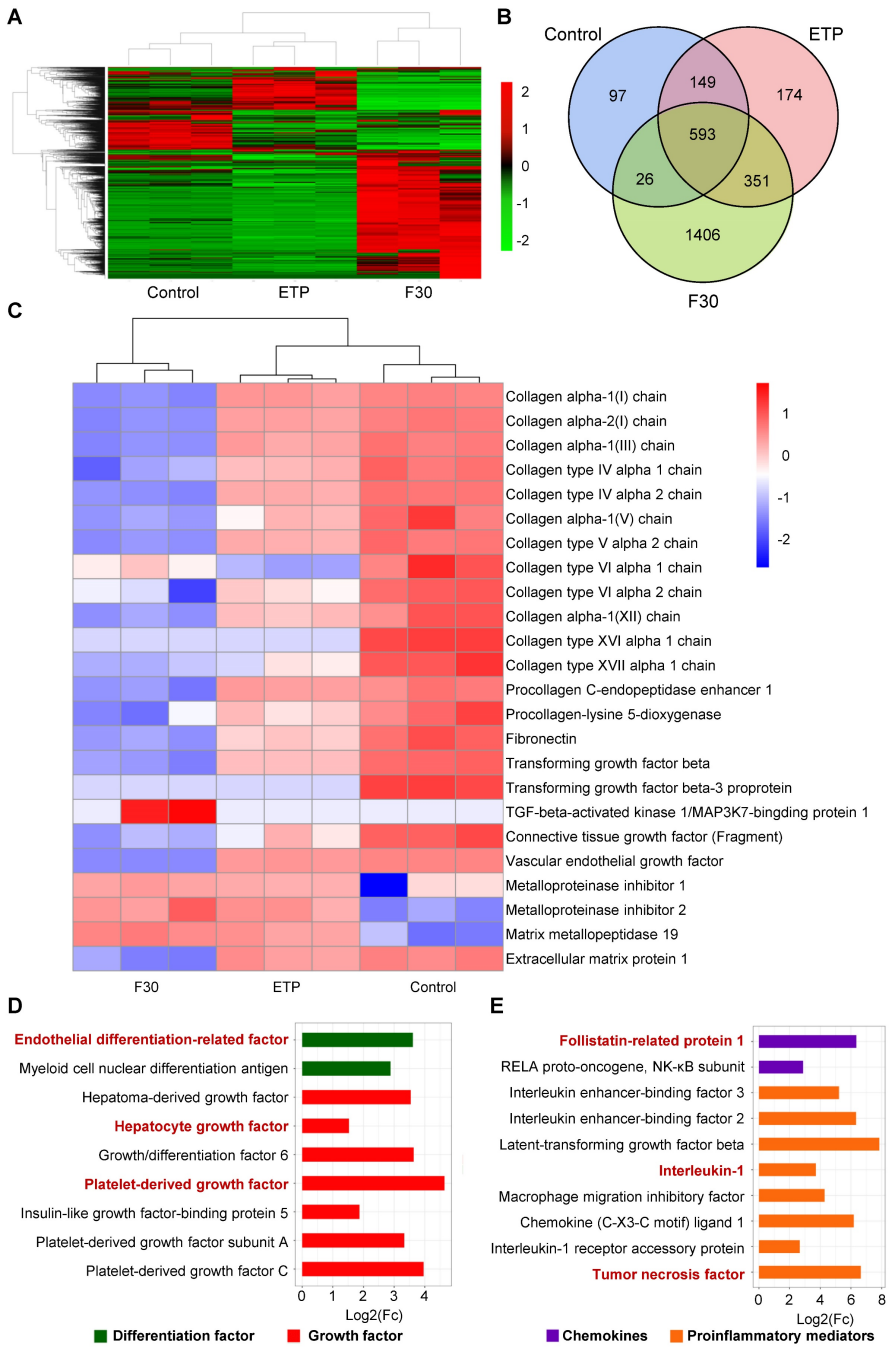
** Secretory profile of senescent HSCs. (A)** Heatmap of secretory proteins of control (growing HSCs with no treatment, n = 3), ETP (n = 3) and F30 (n = 3) HSCs. **(B)** Overlapping results of secretory proteins in indicated groups.** (C)** Heatmap of functional protein associated with activation, collagen synthesis and matrix metallopeptidase. **(D)** Fold change of 9 differentiation and growth factors in F30 in comparison to controls (growing HSCs with no treatment; p < 0.05). **(E)** Fold change of 10 chemokines and proinflammatory mediators in F30 in comparison to controls (p < 0.05).

Label-free quantitative proteomic analysis was performed in ETP-treated and F30-HSCs to further elucidate secretory proteome profiling of senescent HSCs. As shown in **Figure 3A-B**, 2796 kinds of differential proteins were identified through proteomic analysis. Consistent with the qRT-PCR results, collagen proteins and proteins of activated HSC markers were decreased; whilst the secretion of MMP proteins was increased in ETP-treated and F30-HSCs (**Figure 3C**). From a secretory factor perspective, the secretion of differentiation factors and growth factors, such as hepatocyte growth factor (HGF), IGF and PDGF, was significantly increased in senescent HSCs in comparison with the control cells (**Figure 3D**). Several proinflammatory mediators and chemokines, such as IL-1 and tumor necrosis factor (TNF), were also identified from the pool of proteomic data (**Figure 3E**).

### Senescent HSCs secreted proteins favoring hepatocellular malignant transformation

**Figure 4:**
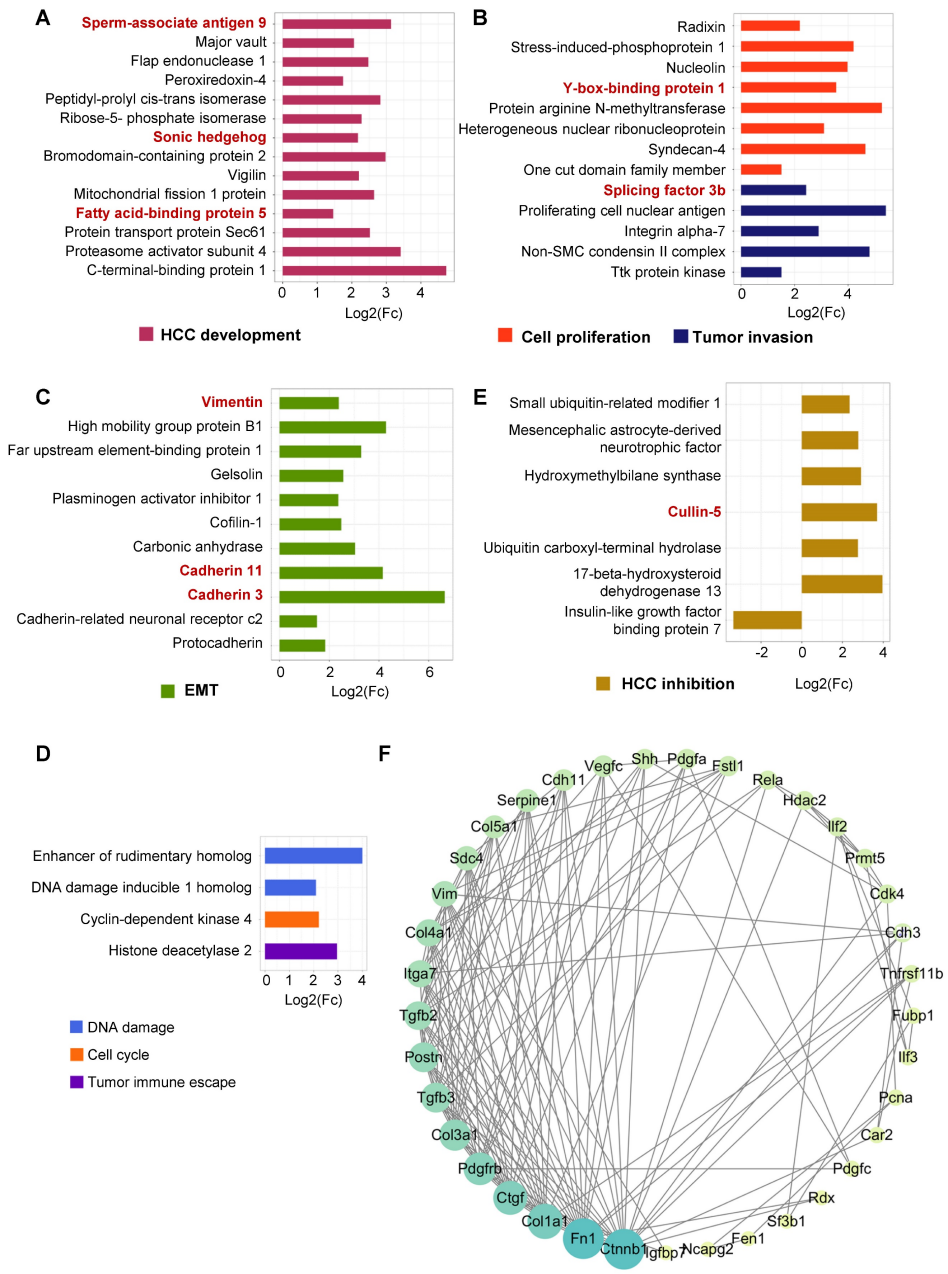
** Secretory proteins favoring hepatocellular malignant transformation in senescent HSCs. (A)** Fold change of 14 secretory proteins in association with HCC development in F30 compared to controls (p < 0.05).** (B)** Fold change of 13 secretory proteins involved in cell proliferation and tumor invasion in F30 compared to controls (p < 0.05). **(C)** Fold change of 11 secretory proteins involved in epithelial-mesenchymal transition (EMT) in F30 compared to controls (p < 0.05).** (D)** Fold change of 4 secretory proteins involved in DNA damage, cell cycle and tumor immune escape in F30 compared to controls (p < 0.05). **(E)** Fold change of 7 secretory proteins contributing to HCC inhibition in F30 compared to controls (p < 0.05).** (F)** Protein-protein interaction network (PPI network) of differential secretory proteins. Size and color shade of the cycle represent the number of interacting proteins. Proteins are sorted according to quantity in clockwise.

In addition to these proinflammatory mediators and chemokines, a variety of proteins that might affect hepatocellular behaviors were found to increase in the secretory proteome profile of senescent HSCs, which were briefly classified into HCC development (**Figure 4A**), tumor invasion (**Figure 4B**), cell proliferation (**Figure 4C**), epithelial-mesenchymal transition (EMT) (**Figure 4C**), tumor immune escape (**Figure 4D**), cell cycle (**Figure 4D**) and DNA damage-related (**Figure 4D**) based on their functionality. For example, sperm-associated antigen-9 affected hepatoma cell growth and metastasis via JNK pathway 27 and fatty acid-binding protein-5 promoted angiogenesis 28 in HCC development. Peroxiredoxin-4, bromodomain-containing protein 2 and Y-box-binding protein 1 potentiated HCC development and tumor invasion via Wnt/β-catenin pathway 29-31. The secretion of two key molecules in EMT, vimentin and cadherin 3/11, was increased, while proteins involved in tumor immune escape, cell cycle and DNA damage were also increased to varying extent. The mechanisms of SASP components capable of interacting with hepatocytes to induce malignant transformation have been listed in **Table S4**. As illustrated in **Figure 4D**, senescent HSCs-derived secretome interacts with a variety of protein components in a complex network. Proteins that might have negative impacts on HCC development and progression were also found to increase in senescent HSCs, such as Cullin-5 that is capable of scavenging proteins required for sustained tumor cell division 32 (**Figure 4E**). However, the quantity of these proteins was much less than those enhancing HCC progression. Insulin-like growth factor-binding protein 7 (IGFBP7) has been identified as a potential tumor suppressor in HCC, and the secretion of IGFBP7 was significantly decreased in senescent HSCs in favor of tumor growth and progression. Based on these differential proteins, protein-protein interaction network (PPI network) was mapped and sorted by the number of potential interactions of each protein (**Figure 4F**). It turned out that β-catenin had the most potential interactions, which is a key protein of Wnt signaling pathway that has been identified as one of the classical signaling pathways in HCC 33. Immunofluorescent and immunohistochemical staining of β-catenin (**Figure S4A-B**), confirming the activation of Wnt signaling pathway in the tumor tissue in this murine NASH-fibrosis-HCC model. Together with all the secretory proteins identified in the proteomic analysis, it was evident that senescent HSCs secreted numerous proteins that largely favor hepatocellular malignant transformation.

### Secretory proteins from senescent HSCs enhanced Hedgehog and Wnt signaling pathways

**Figure 5:**
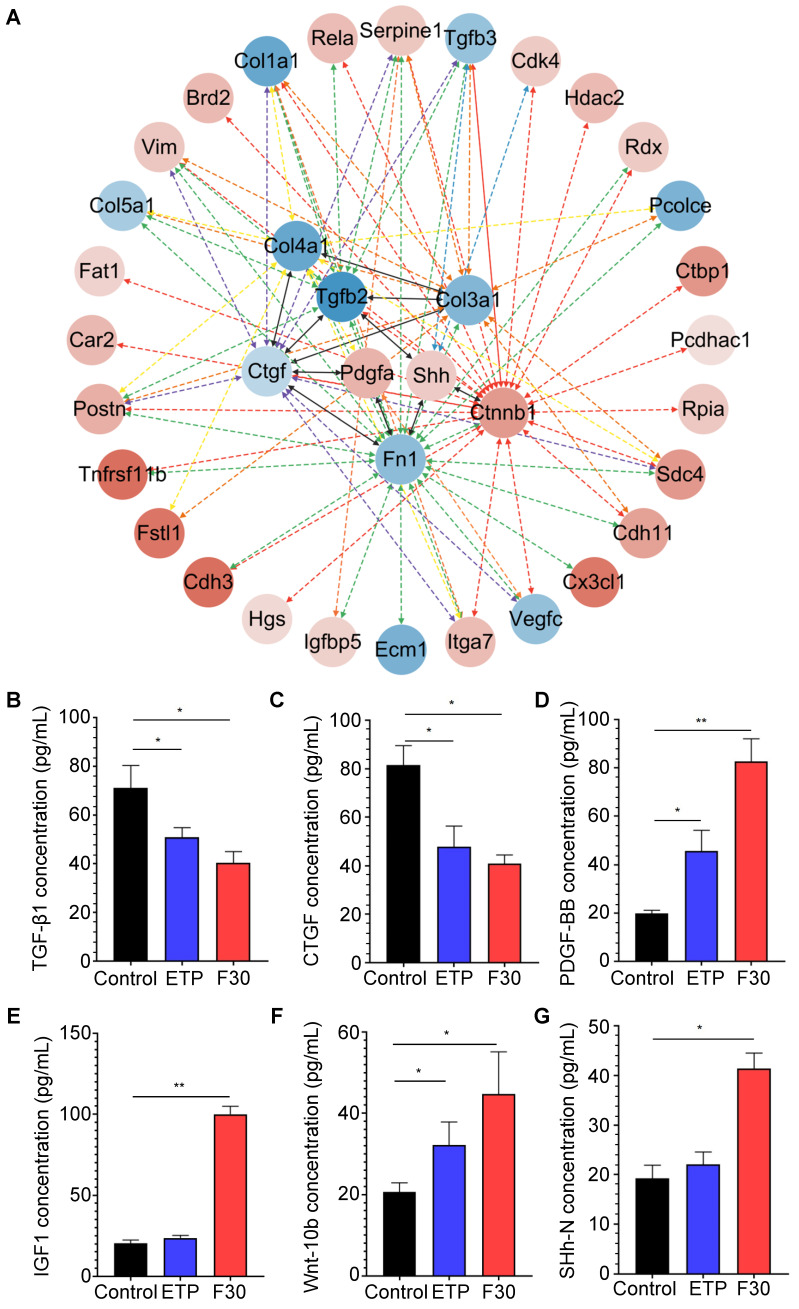
** Key secretory proteins enhanced Hedgehog and Wnt signaling pathway. (A)** PPI (protein-protein interaction) network of secretory proteins enhanced Hedgehog and Wnt signaling pathway. The 8 hits in the middle of the network were key secretory proteins involved in the activation of signaling pathways. Secretory proteins that could directly interact with these 8 key proteins were presented in the outer cycle. Red represents up-regulation of the genes, and conversely blue represents down-regulation. Color gray represents the up/down-regulation levels of genes. **(B)** ELISA-based quantification of TGF-β1 levels in supernatant of control, ETP and F30-HSCs. **(C)** ELISA-based quantification of CTGF levels. **(D)** ELISA-based quantification of PDGF-BB levels. **(E)** ELISA-based quantification of IGF1 levels. **(F)** ELISA-based quantification of Wnt-10b levels. **(G)** ELISA-based quantification of SHh-N levels. All data were expressed as mean ± SEM. *p < 0.05 and **p < 0.01 compared to control (growing HSCs with no treatment).

Although β-catenin was predicted to have the most interactions with other active proteins, β-catenin itself is not a secretory protein. To further investigate critical signaling pathways that promote hepatocellular malignant transformation, the secretory proteins that may directly interact with β-catenin were re-analyzed. As one of major secretary ligands for Hedgehog signaling pathway which has been well documented to play a predominant role in HCC initiation, progression, drug resistance and metastasis in our previous studies 34, 35, sonic hedgehog (SHh) ligand is secreted in both paracrine and autocrine fashions; the transcription factor, Gli-1, was identified to increase in progenitor cells in precarcinogenic dysplastic lesions in the transition from NASH-fibrosis to HCC 16. Of interest, SHh was found to directly interact with a fibrogenic cytokine (TGF-β) and an extracellular matrix component (fibronectin) (**Figure 5A**), and was increased in senescent HSCs (**Figure 4A**). The results of secretory proteome profiling were further verified by ELISA. Consistently, the concentrations of two fibrogenic cytokines, TGF-β1 and CTGF, were decreased in senescent HSC supernatant (**Figure 5B-C**); whereas the concentration levels of PDGF-BB and IGF1 were increased in senescent HSCs-derived medium compared to that from growing HSCs (**Figure 5D-E**). The two ligands of Wnt and Hedgehog signaling pathway Wnt10b and SHh were increased in senescent HSCs (**Figure 5F-G**). In summary, major signaling molecules, such as SHh, β-catenin, Wnt10b, for both Hedgehog and Wnt signaling pathways were significantly increased in senescent HSCs, and may participate in hepatocellular malignant transformation under a steatotic, inflamed and fibrotic microenvironment.

### Hedgehog signaling pathway was activated in HSCs during progression of NASH-HCC

**Figure 6:**
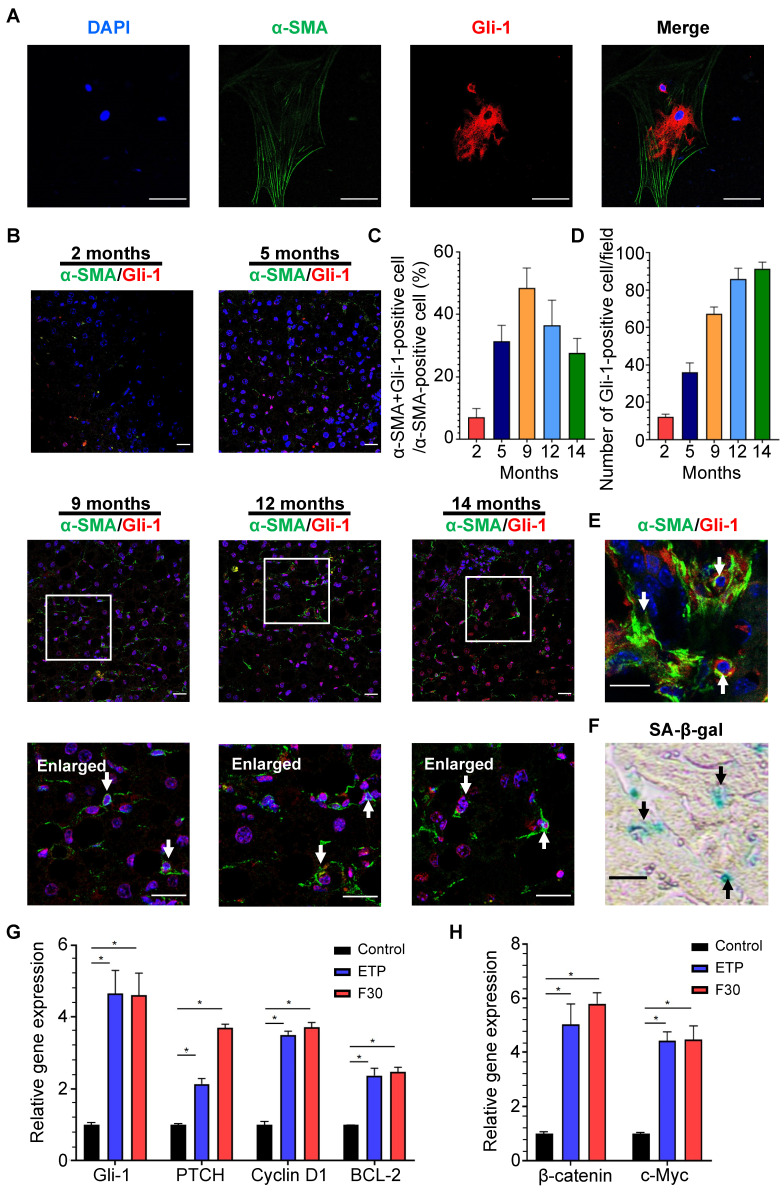
** Activation of Hedgehog signaling pathway in HSCs during progression of NASH-fibrosis-HCC. (A)** Representative micrographs of immunofluorescent staining of α-SMA (green) and Gli-1 (red) in rat primary HSCs. DAPI was used for nucleus staining (blue). Gli-1-positive presented in nucleus indicated the activation of Hedgehog signaling pathway. Images were taken at original magnification (630×). Scale bars = 50 μm. **(B)** Representative micrographs of immunofluorescent staining of α-SMA (green) and Gli-1 (red) in liver sections of HFCD-HF/G diet-fed mice for 2, 5, 9, 12 and 14 months. Images were taken at original magnification (200×). Scale bars = 50 μm. Amplified micrographs were the enlargement of the micrographs at 9, 12 and 14 months above. The co-localization of α-SMA with Gli-1 was indicated by arrow symbols. Amplified images were taken at original magnification (630×). Scale bars = 50 μm. **(C)** The quantitative analysis of Hedgehog signaling pathway in activated HSCs from 2 to 14 months. **(D)** The quantitative analysis of Hedgehog signaling pathway in hepatocytes from 2 to 14 months. **(E)** Immunofluorescent staining of α-SMA (green) and Gli-1 (red) and **(F)** SA-β-Gal staining on two continuous frozen liver sections of HFCD-HF/G-fed mice for 9 months. The same senescent HSCs with activated Hedgehog signaling pathway were indicated by arrow symbols. Images were taken at original magnification (630×). Scale bars = 50 μm. **(G)** Relative mRNA levels of Hedgehog signaling pathway genes (Gli-1, PTCH, cyclin D1 and BCL-2) in senescent HSCs. **(H)** Relative mRNA levels of Wnt signaling pathway genes (β-catenin and c-Myc) in senescent HSCs. All data were expressed as mean ± SEM. *p < 0.05 compared to control.

Accumulation of Hedgehog-responsive myofibroblast-like cells has been found in NAFLD 36, but whether Hedgehog signaling pathway is involved in HSC activation and senescence remains unclear. Through immunofluorescent staining, Gli-1, a key protein of Hedgehog signaling pathway, was found to be present in the cytoplasm and nucleus of rat primary HSCs with α-SMA-positive staining (**Figure 6A**). The same staining was performed on the liver sections of mice fed HFCD-HF/G diet for 2, 5, 9, 12 and 14 months (**Figure 6B**). The activation of Hedgehog signaling pathway in HSCs was initially increased from 5-9 months and then decreased in the late stage of NASH-HCC progression (**Figure 6C**). Consistently with the number of senescent HSCs, Gli-1-positive HSCs ranked highest at 9 months, which further supported a prevailing hypothesis that hedgehog signaling pathway may participate in activation, phenotypic transformation to myofibroblast cells and senescent process after their activation of HSCs in the progression of NASH-HCC.

### Senescent HSCs exhibited elevated activation of Hedgehog signaling pathway

SA-β-Gal and immunofluorescent staining were separately performed on two continuous frozen liver sections from HFCD-HF/G-fed mice for 9 months, the time with the most senescent HSCs (counter-staining of α-SMA with Gli-1). Activation of Hedgehog signaling pathway was found in the same senescent HSC **(Figure 6E-F)**, which indicated that the Hedgehog pathway was kept active from HSC activation to senescence. The expression levels of key signaling molecules of the Hedgehog pathway, such as Gli-1, PTCH, cyclin D1 and BCL-1 were consistently increased in senescent HSCs compared to the controls **(Figure 6G)**. In addition, expression levels of Wnt signaling pathway genes, such as oncogenic β-catenin and c-Myc, were increased in senescent HSCs **(Figure 6H)**, which supported that the activation of both Hedgehog and Wnt signaling pathways was boosted during HSC senescence after activation.

### Senescent HSCs favored hepatocellular malignant transformation through altered secretome

**Figure 7:**
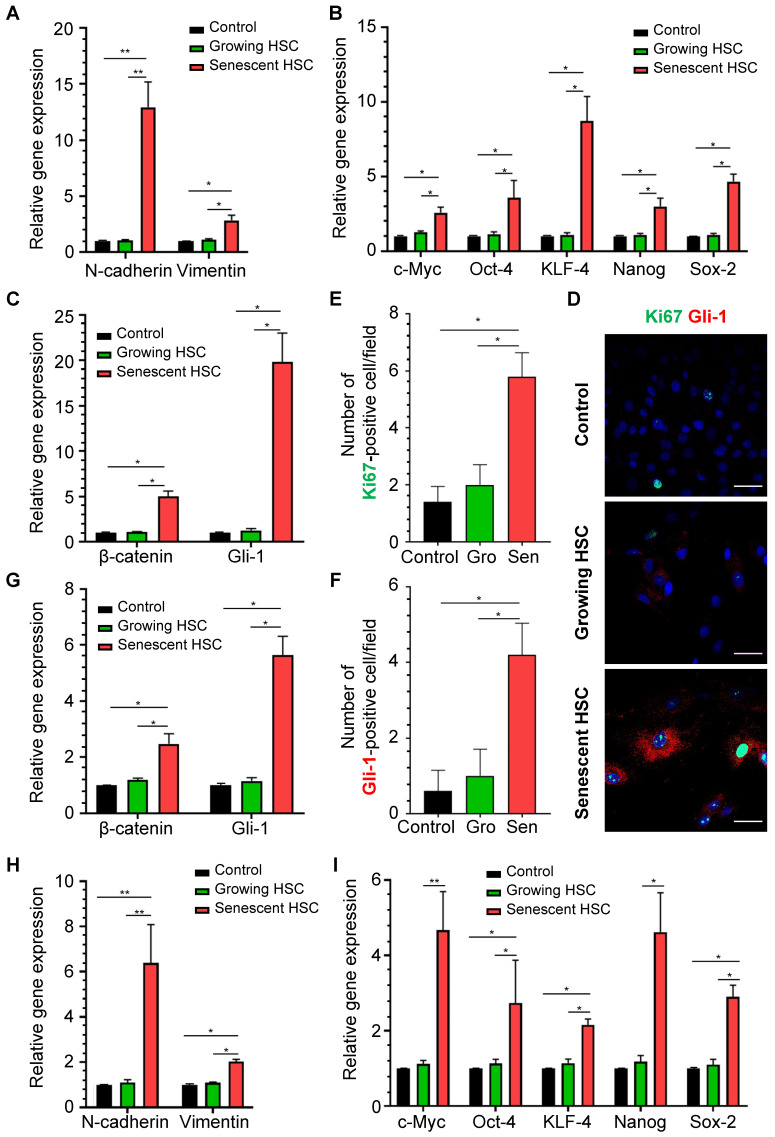
** Effects of medium supernatant from senescent HSC on mouse primary hepatocytes or co-cultured with senescent HSCs. (A)** Relative mRNA levels of epithelial-mesenchymal transition (EMT)-genes (N-cadherin and vimentin) in hepatocytes treated with growing or senescent HSC (F30-HSC) medium supernatant. **(B)** Relative mRNA levels of 5 pluripotent and morphogenic genes in hepatocytes treated with growing or senescent HSC (F30-HSC) medium supernatant. **(C)** Relative mRNA levels of β-catenin and Gli-1 in hepatocytes treated with growing or senescent HSC (F30-HSC) medium supernatant. **(D)** Immunofluorescent staining of Ki67 (green) and Gli-1 (red) in hepatocytes treated with growing or senescent HSC (F30-HSC) medium supernatant. Images were taken at original magnification (630×). Scale bars = 50 μm. (**E**) The quantitative analysis of Ki67-positive hepatocytes. **(F)** The quantitative analysis of Gli-1-positive hepatocytes. Gro = Growing. Sen = Senescent. All data were expressed as mean ± SEM. *p < 0.05 and **p < 0.01 compared to controls (no treatment) or growing HSC medium supernatant. **(G)** Relative mRNA levels of β-catenin and Gli-1 in hepatocytes co-cultured with growing or senescent HSCs (F30-HSC). (**H**) Relative mRNA levels of EMT-related genes (E-cadherin, N-cadherin and vimentin) in hepatocytes co-cultured with growing and senescent HSCs (F30-HSC). **(I)** Relative mRNA levels of 5 pluripotent and morphogenic genes in hepatocytes co-cultured with growing or senescent HSCs (F30-HSC). All data were expressed as mean ± SEM. *p < 0.05 and **p < 0.01 compared to controls (hepatocytes cultured alone) or co-cultured with growing HSCs.

To determine whether senescent HSC secretory proteins could really affect hepatocellular behaviors, primary mouse hepatocytes were treated for 48 h with the supernatant of growing HSCs and F30-HSCs to evaluate expression levels of the genes involved in hepatocellular EMT, pluripotency and morphogenesis and Hedgehog signaling pathways which are critical for the initiation of carcinogenic transformation and malignant progression. Expression levels of EMT-associated genes, such as N-cadherin and vimentin, were significantly increased in primary hepatocytes treated with medium supernatant from senescent HSC culture (**Figure 7A**). However, the medium supernatant from growing HSCs seemed to have no significant effects on these genes from primary hepatocytes. Expression levels of pluripotent genes; including c-Myc, Oct-4, KLF-4, Nanog and Sox-2, were remarkably increased in hepatocytes treated with medium supernatant from senescent HSCs when compared to that from growing HSCs (**Figure 7B**).

These pluripotent and morphogenic (Gli-1) genes are considered to be critical for development of dysplasia or further transformation to malignancy in a murine NASH-HCC model as reported by us 16. Immunofluorescent staining of Gli-1 and Ki67 was performed on the mouse primary hepatocytes after stimulation from senescent HSC-conditioned medium (**Figure 7D**). Numbers of both Gli-1- and Ki67-positive cells were increased in hepatocytes treated with senescent HSC-conditioned medium when compared to controls or growing HSCs (**Figure 7E-F**). Immunofluorescent staining of β-catenin revealed the activation of Wnt signaling pathways in hepatocytes (**Figure S4C**), which indicated that activation of Hedgehog and Wnt signaling pathway and proliferation of mouse primary hepatocytes were boosted after stimulation from senescent HSC-conditioned medium.

Expression levels of β-catenin and Gli-1 in senescent supernatant-treated hepatocytes were higher than the supernatant from growing HSCs** (Figure 7C)**, which supported the notion that the secretory proteins from senescent HSCs facilitated activation of Hedgehog and Wnt signaling pathways in neighboring hepatocytes. Given that the immunofluorescent staining of Gli-1 on mouse liver sections showed increasing activation of Hedgehog signaling pathway in hepatocytes in the progression of NASH-HCC (**Figure 6D**), the altered secretory protein profile from senescent HSCs might be essential factors contributing to sustained activation of Hedgehog signaling pathway in driving steatotic or dysplastic hepatocytes towards malignant transformation.

**Figure 8:**
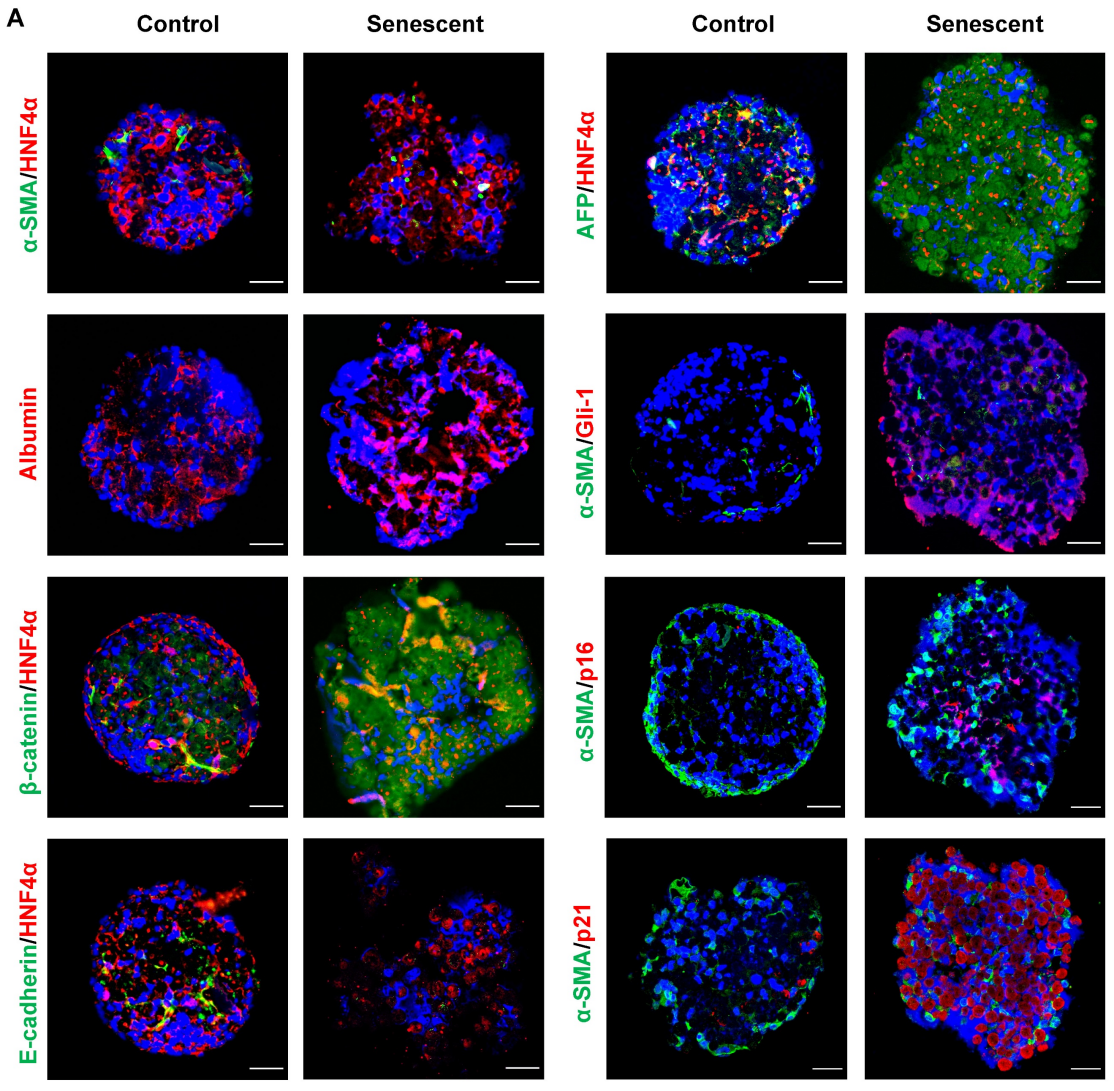
** Co-culture in 3D spheroids of senescent HSCs with rat primary hepatocytes. (A)** Representative micrographs of immunofluorescent staining of HNF4α (hepatocyte markers, red) with β-catenin, E-cadherin and AFP (green) or α-SMA (HSC marker, green) with Gli-1 (Hedgehog pathway marker, red), p16 and p21 (senescent cells marker, red) in 3D spheroids after 2 weeks. Images were taken at original magnification (630×). Scale bars = 50 μm.

To further confirm the effects of senescent HSCs on primary hepatocytes, mouse primary hepatocytes were co-cultured with growing or senescent HSCs in transwells for 24 h, and mRNA levels of most genes mentioned above were determined. Similarly, gene expression of β-catenin and Gli-1 was increased (**Figure 7G**) and so was the expression of EMT (**Figure 7H**) and pluripotent and morphogenic genes in primary hepatocytes when co-cultured with senescent HSCs in comparison with control HSCs (**Figure 7I**). In order to further explore the effects on senescent HSCs on primary hepatocytes for longer term, 3D spheroids of co-culture of senescent HSCs with primary hepatocytes were used for validation (**Figure 8A**). Activated (control group) or 30-passage-induced senescent HSCs (senescent group) with primary hepatocytes were assembled in 3D spheroids. After co-culture in close contact for 2 weeks, the results revealed increased levels of α-fetoprotein (AFP), β-catenin, and Gli-1 in the senescent group compared to the control group. Additionally, the cellular state (loss of epithelial characteristics) of the 3D spheroids in senescent group was found to be worse than that of the control group as indicated by less expression of E-cadherin (**Figure 8A**). Taken together, these findings further demonstrated that altered senescent HSC secretory proteins favored hepatocellular malignant transformation with increased levels of pluripotent, morphogenic and oncogenic factors, although the direct* in vivo* evidence remains to be attained.

## Discussion

Senescence has been identified as one fate of activated HSCs and could limit liver fibrosis in murine models of hepatic fibrosis caused by CCl_4_ intoxication 37 or cholic acid toxicity 15, however the role of these senescent HSCs in the malignant transformation from NASH to HCC remains elusive. Two recent studies have implicated HSC senescence in the favor of HCC development 15, 38. Suppression of HSC senescence via p53 deletion resulted in M2 macrophage polarization and proliferation of premalignant hepatocytes 38; and SASP, such as DCA, promoted HCC development in obesity-related microenvironment 15. In the present study, senescent myofibroblast-like cells/HSCs were identified in NASH-HCC specimens and a well-characterized murine NASH-fibrosis-HCC model 16. A full spectrum of senescent HSC secretory protein components was identified through label-free quantitative proteomic (NanoRPLC-MS/MS) analysis. Moreover, the release of critical factors was quantitatively verified by ELISA, and their direct actions on hepatocellular behaviors, such as proliferation and EMT, were investigated by co-cultures of primary hepatocytes or 3D co-culture spheroids with senescent HSCs. The findings demonstrate that secretory proteins from senescent HSCs drove hepatocytes to undergo phenotypic changes along a malignant direction, to manifest with EMT, pluripotent and morphogenic profiles and enhanced proliferation, towards de-differentiation *in vitro*.

To *in vivo* trace dynamic changes of senescent HSCs in chronic liver injury possesses technical barriers, and only snapshots of SA-β-Gal-positive cells can be counter-stained in α-SMA-positive cells at different time points of animal experiments or biopsied specimens. Therefore, *in vitro* models of senescent HSCs were used to investigate the fate of activated HSCs and the impacts of senescent HSCs on the behaviors of other cell types, such as hepatocytes. The choice of rat immortalized BSCC-10 HSCs to induce the senescence *in vitro* instead of other immortalized cell lines is based on the mainstream mechanism of cell senescence, i.e. telomere crisis. Unlike other immortalized HSC lines that artificially maintain telomere length by transfection with the telomerase reverse transcriptase (hTERT) gene or large T antigen 19, 20, BSCC-10 cells exhibited spontaneous immortalization 17, eliminating the controversy about telomerase length in senescent cells. Two methods, i.e. ETP and serial passages, were employed to induce senescent cells, and each method has its own features. ETP enables to gain senescent cells quickly; in contrast, serial passages up to 30 generations took a longer duration than the former one. As demonstrated in the secretory protein profile, it appeared that ETP-induced senescent HSCs did not differ dramatically from the F30-HSCs in the gene expression results. Given that ETP induced HSCs to be SA-β-Gal-positive through DNA damage, this approach might not be sufficient enough to provoke cells to exhibit a complete senescent phenotype in a short time frame. Therefore, in this context, serial passages may be preferential as a more reliable approach to induce senescence in HSCs in contrast to EPT treatment.

One of striking phenomena in the complicated interactions is that SASP components actually interact with Hedgehog and Wnt signaling pathways. Therefore, it is attempting to assume that senescent HSC secretory proteins may directly activate the Hedgehog and Wnt signaling pathways in neighboring hepatocytes in a paracrine fashion. In addition, SHh activates Hedgehog signaling pathway in hepatocytes in the form of autocrine, thus leads to a self-amplifying loop of Hedgehog activation and ligand release, and the ligand acts on steatotic or dysplastic hepatocytes for further transformation to malignancy.

Increasing studies have implicated an array of impacts of liver fibrosis on HCC initiation and progression. The fibrotic environment might be favorable for the development of HCC, as fibrosis creates a hypoxic condition which stimulates angiogenesis 39. Deposition of ECM components, such as fibronectin, in fibrotic liver facilitated the responses of endothelial cells to growth factors, such as HGF, thus enhanced cell migration and proliferation, which are crucial for neovascularization 40. As effector cells in hepatic fibrogenesis, HSCs play an important role in HCC development, which has been a key attention in the delineation of how fibrosis acts as a precarcinogenic niche for hepatic carcinogenesis. HCC xenograft tumors grew quicker in a fibrotic than in normal liver 41. In addition to the secretion of angiogenic factors, such as VEGF and CXC chemokines, which stimulate tumor vascularization by activated HSCs 42, 43, the secretion of cytokines and chemokines, such as PDGF, TGF-β, IL-6 and Wnt ligands, might directly interact with hepatic cells to initiate the HCC development or facilitate its progression 41, 44. Moreover, the pro-inflammatory cytokines released by HSCs affects the microenvironment, and expression of PD-L1 might induce exhaustion of T lymphocytes 45, thus enable malignant cells escaping from immune surveillance and attack. All of these provide a specific environment that is conducive to the survival of pre-malignant cells and further transformation to hepatic malignancy.

Growing evidence has supported that the senescence of HSCs may be favorable for the development of HCC, however, there is no direct evidence to validate this assumption. An inflamed and fibrotic liver creates a microenvironment favoring tumor cell survival and growth; whereas active protein components released from activated and senescent HSCs are critical factors triggering for malignant transformation of steatotic, degenerated or dysplastic hepatocytes. These pathologic alterations of hepatocytes were well characterized in NASH-fibrotic progression in transition to HCC in our previous study 16. The activation of transcription factors, such as c-Myc, KLF-4, and Nanog secreted in these pre-malignant cells function as the internal drivers for oncogenic or pluripotent fate changes 16. It is clear that expression of these oncogenic transcription factors and secretion of the proteins were significantly increased in senescent HSCs than activated HSCs in the present and previous studies. Presumably, the SASPs of senescent HSCs amplify the effects of both external and internal factors; and thus accelerate the process of hepatocellular malignant transformation in an inflamed and fibrotic microenvironment. In addition, senolytic treatment has been reported to be effective in inhibiting HCC progression in an approach of depleting senescent HSCs, however the treatment was ineffective in improving hepatic fibrosis during a fibrotic stage 46, which suggested that hepatic fibrosis is insufficient to drive HCC, instead sustained senescence of activated HSCs may be required in the development of HCC.

Induction of apoptosis and senescence of activated HSCs are two therapeutic goals in alleviating hepatic fibrosis. Several approaches were reported to induce HSC senescence in order to attenuate liver fibrosis 8, 10. A major emphasis has been focused on the successful induction of senescent HSCs; however little attention has been paid to whether these senescent HSCs left *in situ* might exert impacts other than therapeutic effects or even pro-malignant effects. Although *in vivo* transformation from NASH-fibrosis to malignancy is much more complex than *in vitro* models, such as co-culture or 3D spheroids, the findings from the present study underscore the critical role of senescent HSCs in this process, and implicate potential molecular targets in the prevention of progression of NASH to fibrosis and further to malignant transformation. Therefore, therapeutic approaches aiming at inducing HSC senescence might be a double-edged sword, alleviating fibrosis while possibly enhancing the risk of HCC development. In this context, etiologic elimination or early intervention would be ideal for chronic liver injury in order to reduce fibrogenic responses by suppressing HSC activation.

In spite of the novel findings from the present study, obviously there are two main limitations to disclose. The first, lack of a key animal model. Labeling senescent cells in animal models remains a challenge in the field of cell senescence research 47. The absence of targeting approaches to precisely induce senescence of a specific cell type *in vivo*, such as HSCs and the lack of tracking techniques to *in situ* observe individual cells from activation to senescence in a manner of longevity make it difficult to establish key animal models. Moreover, inducing HSC senescence while promoting the progression of HCC in a murine NASH model makes the validating process extremely challenging. Alternatively, an *in vitro* co-culture of 3D spheroids of primary hepatocytes with senescent HSCs was utilized for validation with a longer term in a close-contact pattern (**Figure 8**). The second, the lack of probes to *in situ* label senescent cells also makes it challenging in directly isolating primary senescent HSCs from a murine fibrotic model. Therefore, the results from *in vitro* experiments are solely based on ETP-treated or 30F-passage-induced senescent cell lines due to the technical challenges mentioned above.

In conclusion, increased senescent HSCs were identified in NASH-fibrotic progression and HCC specimens, and SASPs from senescent HSCs play a critical role in affecting behavior changes in neighboring hepatocytes through proliferation, EMT or migration, and may activate oncogenic Hedgehog or Wnt signaling pathways to accelerate malignant transformation of degenerated or dysplastic hepatocytes in a steatotic, inflamed or fibrotic microenvironment. The findings of the present study underscore that targeting activated HSCs is critical for regression or reversal of hepatic fibrosis in chronic injury, which may progress to the end-stage liver disease and fatal liver malignancies.

## Supplementary Material

Supplementary figures and tables.

## Abbreviations

AFP: α-fetoprotein
α-SMA: smooth muscle α-actin
BCL-1: B-cell lymphoma-1
CCR: chemokine C-C motif receptor
CTGF: connective tissue growth factor
CXCL: chemokine C-X-C motif ligand
DCA: deoxycholic acid
ECM: extracellular matrix
ELISA: enzyme-linked immunosorbent assay
EMT: epithelial-mesenchymal transition
FGF: fibroblast growth factor
Gli-1: glioma-associated oncogene homolog-1
HBV: hepatitis B virus
HCC: hepatocellular carcinoma
HCV: hepatitis C virus
HFCD-HF/G: high fat/calorie diet plus high fructose/glucose in drinking water
HGF: hepatocyte growth factor
HSC: hepatic stellate cell
hTERT: human telomerase reverse transcriptase
IGF: insulin-like growth factor
IGFBP7: insulin-like growth factor-binding protein-7
IL: interleukin
KLF-4: kruppel-like factor 4
LC-MS/MS: liquid chromatography-mass spectrometry
MMPs: matrix metalloproteinases
MSC: mesenchymal stem cell
NAFLD: nonalcoholic fatty liver disease
NASH: nonalcoholic steatohepatitis
OA: oleic acid
OCT-4: octamer binding transcription factor-4
PA: palmitic acid
PDGF: platelet-derived growth factor
PPI: protein-protein interaction
PTCH: patched protein homolog-1
SA-β-Gal: senescence-associated β-galactosidase
SASP: senescence-associated secretary phenotype
SHh: sonic Hedgehog
TGF: transforming growth factor
TIMP1: tissue inhibitor of metalloproteinase-1
TNF: tumor necrosis factor
