# Supplementary material for: Secretome of senescent hepatic stellate cells favors malignant transformation from nonalcoholic steatohepatitis-fibrotic progression to hepatocellular carcinoma
Source: Theranostics. 2023 Aug 6;13(13):4430–48. doi: 10.7150/thno.85369 (PMC10465212; doi:10.7150/thno.85369)
Supplement: Supplementary file 1 — Supplementary figures and tables. [file thnov13p4430s1.pdf]

Secretome of senescent hepatic stellate cells favors malignant transformation from nonalcoholic steatohepatitis-fibrotic progression to hepatocellular carcinoma

Yuan Zhou[1], Li Zhang[1], Yue Ma[1], Li Xie[1], Yong-yu Yang[1], Cheng Jin[1], Hui Chen[1], Ying Zhou[3], Jia Ding[2], Guang-qi Song[3], Jian Wu[1,4,5]

[1]Department of Medical Microbiology & Parasitology, MOE/NHC/CAMS Key Laboratory of Medical Molecular Virology, School of Basic Medical Sciences, Fudan University, Shanghai 200032, China

[2]Department of Gastroenterology, Shanghai Jing'an District Central Hospital, Fudan University, Shanghai 200040, China;

[3]Joint Laboratory of Biomaterials and Translational Medicine, Puheng Technology Co., Ltd, Suzhou 215163, China

[4]Department of Gastroenterology & Hepatology, Zhongshan Hospital, Fudan University, Shanghai 200032, China

[5]Shanghai Institute of Liver Diseases, Fudan University Shanghai Medical College, Shanghai 200032, China

CORRESPONDING AUTHORS:

Jian Wu, MD, PhD, FAASLD
Department of Medical Microbiology, Key Laboratory of Medical Molecular Virology, School of Basic Medical Sciences
Fudan University Shanghai Medical College
138 Yixue Yuan Road, P. O. Box 228,
Shanghai 200032, China
Tel: +86(21)5423-7705
Fax: +86(21)6422-7201
Email: jian.wu@fudan.edu.cn
ORCID: 0000-0001-9933-7364

Guang-qi Song, PhD
Joint Laboratory of Biomaterials and Translational Medicine
Puheng Technology Co., Ltd
Suzhou 215000, China
song.guangqi@zs-hospital.sh.cn
ORCID: 0000-0002-2409-0771

Jia Ding, MD, PhD
Department of Gastroenterology
Shanghai Jing'an District Central Hospital,
Fudan University
Shanghai 200040, China
Email: jiading12@fudan.edu.cn
ORCID: 0000-0002-7391-8520

SUPPLEMENTARY MATERIALS

SUPPLEMENTARY FIGURES

Figure S1

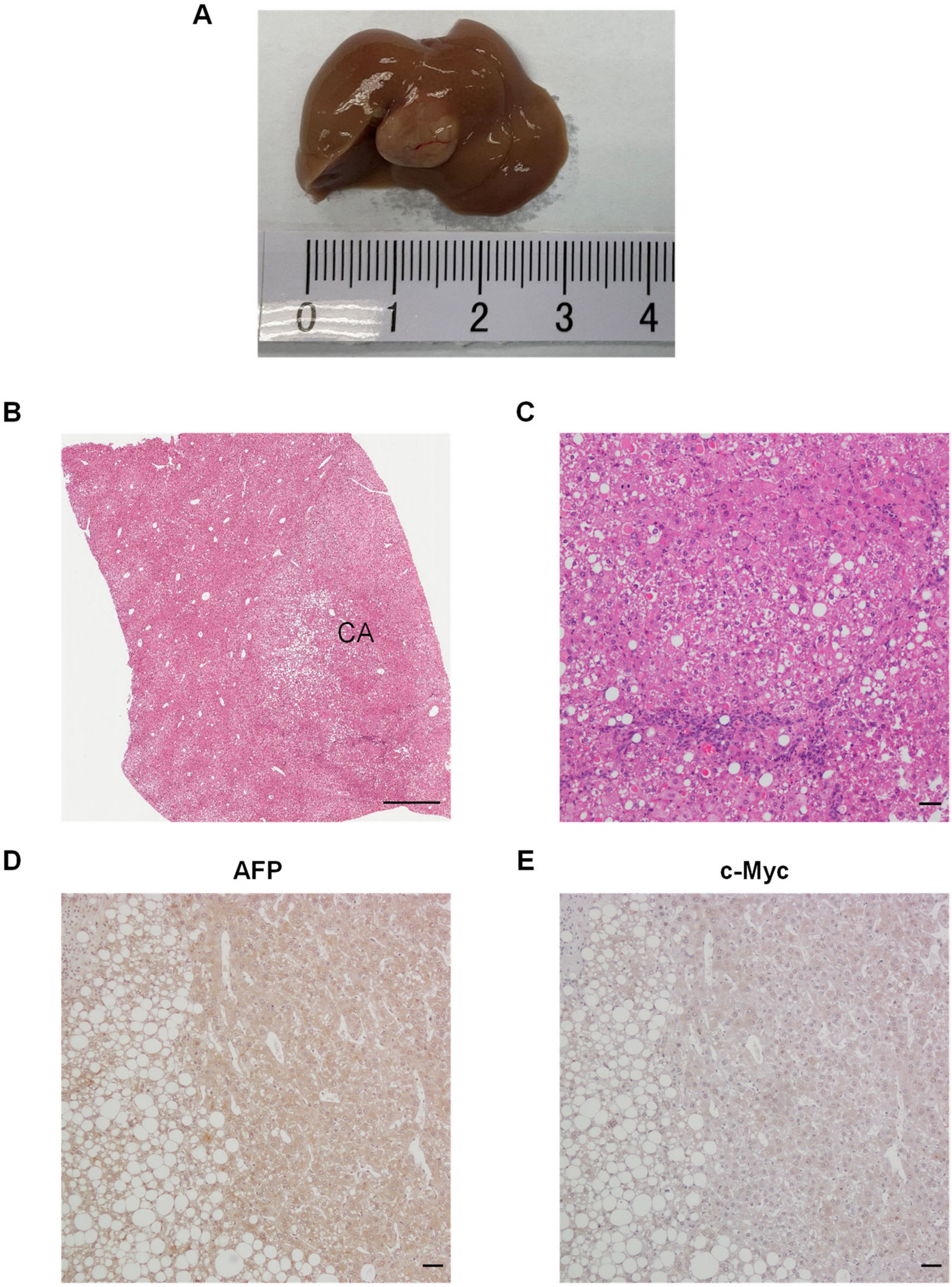

Figure S1 Progression from NASH, fibrosis to HCC in HFCD-HF/G-fed mice at 14 months. (A) Photographs of a liver tumor nodule at 14 months. **(B)** Representative micrographs of H&E staining of tumor nodular tissues. Image was taken at original magnification (20×). Scale bars = 1 mm. **(C)** Representative micrographs of H&E staining of tumor nodular tissues. Image was taken at original magnification (400×). Scale bars = 50 μm. Representative micrographs of immunohistochemical staining of **(D)** AFP and **(E)** c-Myc in tumor tissues of HFCD-HF/G diet-fed mice for 14 months. Images were taken at original magnification (400×). Scale bars = 50 μm.

Figure S2

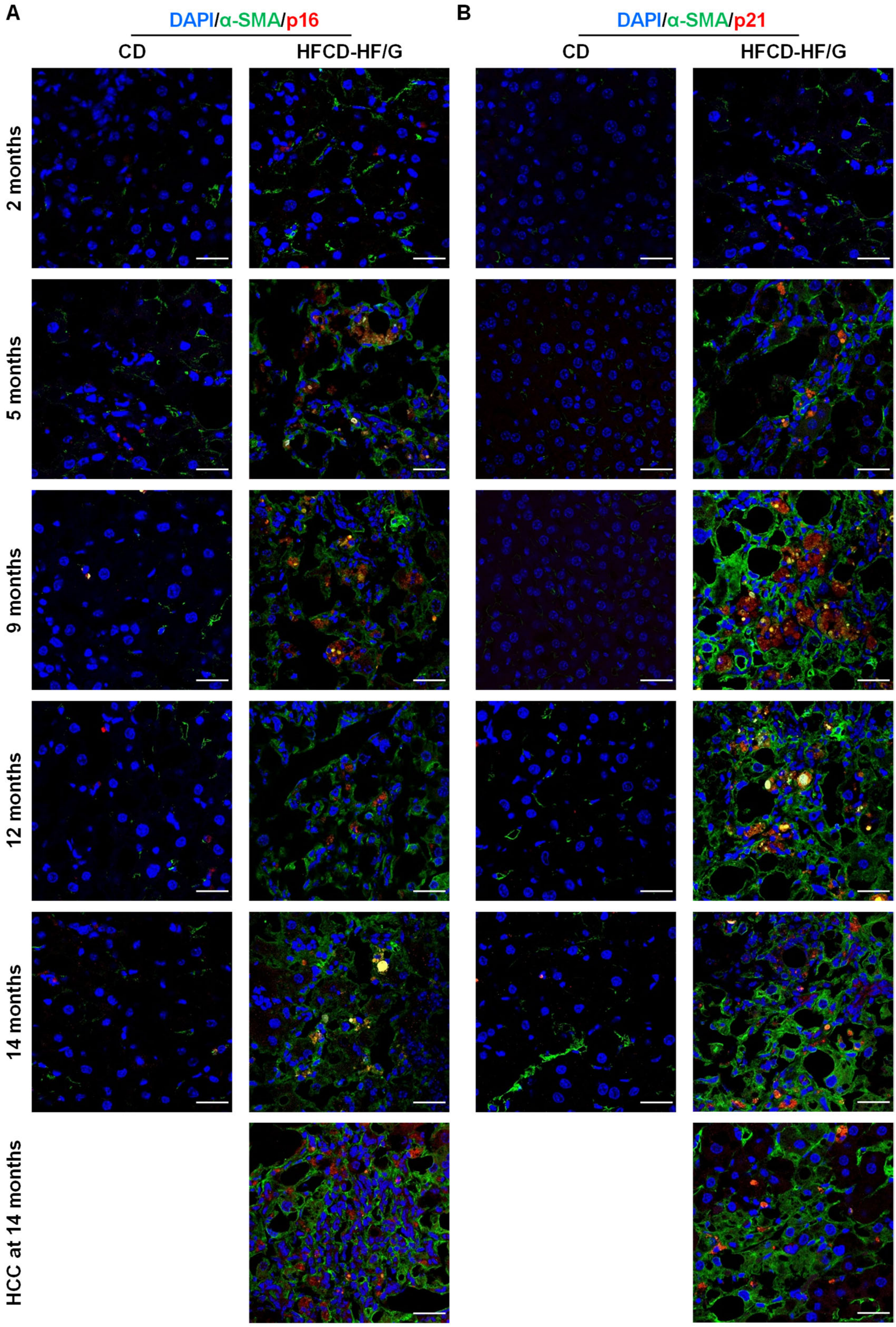

Figure S2 Representative micrographs of p16 and p21 immunofluorescent staining of liver sections from mice NASH-HCC model. (A) Representative micrographs of counter-staining of p16 (red) or **(B)** p21 (red) with α-SMA (green) HSCs of liver tissues

at 2, 5, 9, 12 and 14 months in the control diet or HFCD-HF/G diet-fed mice. Images were taken at original magnification (630×). Scale bars = 50 μm.

Figure S3

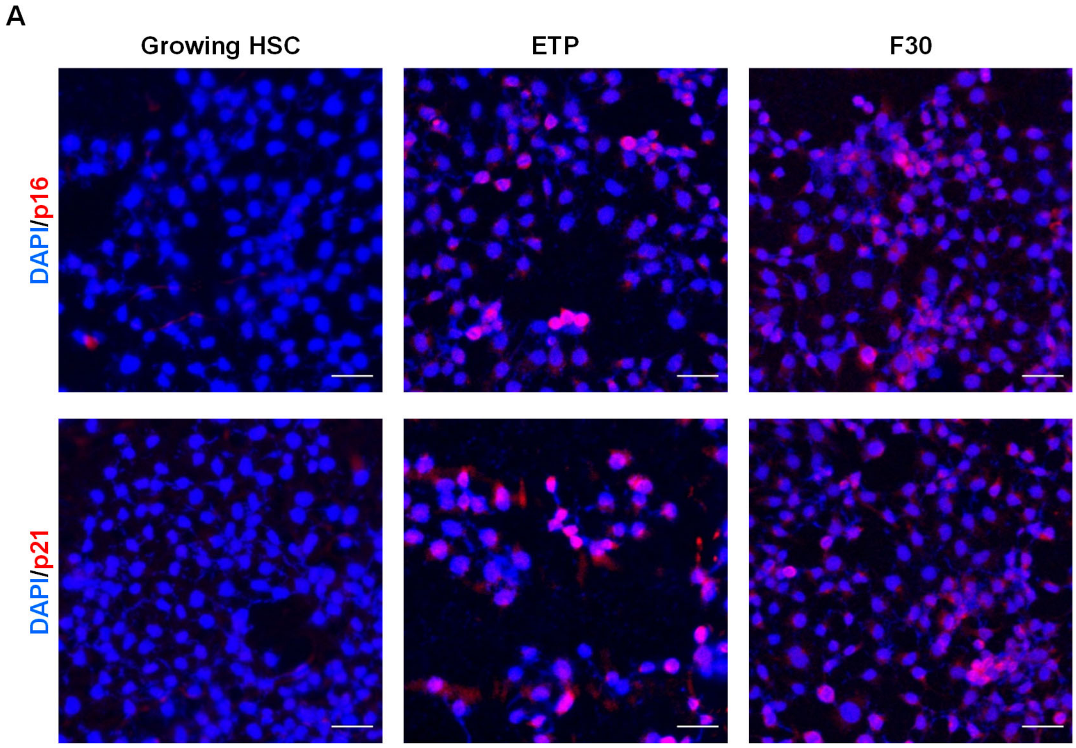

Figure S3 Positivity of p16 and p21 in ETP-treated or 30-passage-induced senescent HSCs. (A) Representative micrographs of fluorescent staining of p16 or p21 positivity in ETP-treated and F30 senescent HSCs. Nuclear location of p16 or p21 was stained in red with counter-staining of nuclei with DAPI. Images were taken at original magnification (200×). Scale bars = 50 μm.

Figure S4

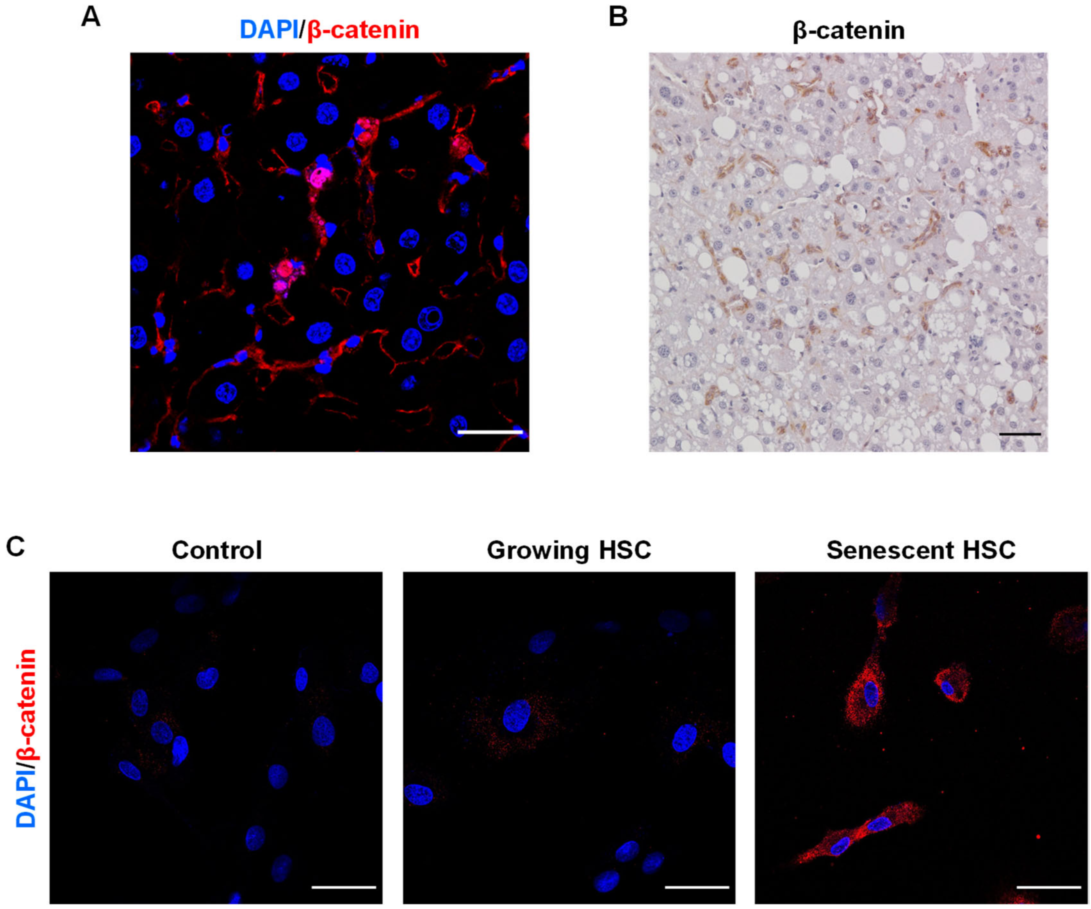

Figure S4 Activation of β-catenin in HCC tissue and mouse primary hepatocytes. Representative micrographs of **(A)** immunofluorescent staining and **(B)** immunohistochemical staining of β-catenin in tumor tissues of HFCD-HF/G diet-fed mice for 14 months. Immunofluorescent staining images were taken at original magnification (630×). Immunohistochemical staining images were taken at original magnification (400×). Scale bars = 50 μm. **(C)** Representative micrographs of immunofluorescent staining of β-catenin in mouse primary hepatocytes treated with growing and senescent HSC (F30-HSC) medium supernatant. Images were taken at original magnification (630×). Scale bars = 50 μm.

SUPPLEMENTARY TABLES

Table S1 Primers used in qRT-PCR

Sequences of mouse primers used in qRT-PCR

Genes	Forward Primer	Reverse Primer
c-Myc	TTGGAAACCCCGCAGACAG	TCTCTTCCTCGTCGCAGATG
Oct-4	TGGGCTAGAGAAGGATGTGGT	GGAAAGGTGTCCCTGTAGCC
KLF-4	TGGGGGTTTTGGTTTGAGGT	ACTGGTGCTGAGCCCTGAATC
Sox-2	AGGAAAGGGTTCTTGCTGGG	ACGAAAACGGTCTTGCCAGT
E-cadherin	AACCCAAGCACGTATCAGGG	ACTGCTGGTCAGGATCGTTG
N-cadherin	GGCCTTGCTTCAGGCGT	CATTGAGAAGGGGCTGTCCT
Vimentin	TTTGCTGACCTCTCTGAGGC	CTCCAGGGACTCGTTAGTGC
β-catenin	GTCAGTGCAGGAGGCCGA	CTCCATCAGGTCAGCTTGAGT
Gli-1	CCATTGGTACCATGAGCCCT	AGCATCATTGAACCCCGAGT
β-actin	GTCAGAAGGACTCCTATGTG	ACGCAGCTCATTGTAGAAG

Sequences of rat primers used in qRT-PCR

Genes	Forward Primer	Reverse Primer
TGF-β1	AGGAGACGGAATACAGGGCT	ACGTTTGGGACTGATCCCATT
CTGF	GCGCCTGTTCTAAGACCTGT	TGCACTTTTTGCCCTTCTTAATGT
TIMP1	CCTCTGGCATCCTCTTGTTG	GGGAACCCATGAATTTAGCC
Procoll-I	GGAAGCGAAGGTTCCGAATC	GCTGTTCTTGCAGTGATAGGTGA
Procollagen III	GCCTACATGGATCAGGCCAA	CACCAGTGTGTTTAGTGCAGC
Procollagen IV	CCCAAAGGCATCAGGGGAAT	ATCCTGGTAAACCAGCCAGC
Fibronectin	CCACCATCACTGGTCTGGAG	GGGTGTGGAAGGGTAACCAG
MMP3	CCTCGTGGTACCCACCAAAT	TTTCGCCAAAAGTGCCTGTC
MMP10	CAATCCCTGTATGGAGCCCG	TCTCAGCATGGTGACTGCAT
MMP12	ACCAGAGCCACACTATCCCA	CTGCCTCACATCGTACCTCC
IL-6	AAGTCCGGAGAGGAGACTTCA	TTGCCATTGCACAACTCTTTT
IL-1β	TCTGTGACTCGTGGGATGAT	TTGTTGTTCATCTCGAAGCC
CXCL1	TGCACCCAAACCGAAGTCAT	ACTTGGGGACACCCTTTAGC
CXCL9	CACTGTGGAGTTCGAGGAACC	GTTAGGGCTTGGGGCAAACT
Gli-1	AACTCCACGAGCACACAGG	TACTCAGCACCAGCATCACC
PTCH	GGGGCTCCGGGAAATTAATAAAAG	CCAGTAGCCTTCCCCATAGCC
Cyclin D1	GTGCCATCCATGCGGAA	GGATGGTCTGCTTGTTCTC
BCL-2	GTCATGTGTGTGGAGAGCGT	ACAGTTCCACAAAGGCATCC
β-catenin	GAAAATGCTTGGGTCGCCAG	CATTTTCTGCAGCCCACCAG
β-actin	AGCTGTGCTATGTTGCCCTA	GAACCGCTCATTGCCGATAG

Table S2 Commercial sources for antibodies used in immunofluorescent staining

Antibody	Company	Species	Catalogue #
α-SMA	CST	Rabbit	19245
α-SMA	Proteintech	Mouse	67735-1-Ig
Gli-1	Proteintech	Mouse	66905-1-Ig
Ki67	Servicebio	Rabbit	GB111141
Albumin	Abgent	Rabbit	P02768
AFP	Proteintech	Rabbit	14550-1-AP
β-catenin	Abmart	Mouse	M24002M
β-catenin	Abclonal	Rabbit	A19657
E-cadherin	Abclonal	Rabbit	A22850
c-Myc	Abways	Rabbit	CY5150
p16	Proteintech	Rabbit	10883-1-AP
p21	Proteintech	Rabbit	10355-1-AP
HNF4α	Abcam	Mouse	ab41898

Table S3 Commercial sources for ELISA kits used for determination of protein concentration in culture medium

Protein	Company	Species	Catalogue #
TGF-β1	Abclonal	Rat	RK00059
CTGF	Abcam	Rat	ab275897
PDGF-BB	Elabscience	Rat	E-EL-R0537c
IGF1	Abclonal	Rat	RK03737
Wnt10b	Novus	Rat	NBP3-00485
Shh-N	R&D	Rat	DSHH00

Table S4 SASP capable of interacting with hepatocytes to induce malignant transformation.

Protein name	Gene name	Function according to reference
Sperm-associated antigen 9	Spag9	Aberrant expression promotes HCC tumorigenesis via JNK pathway [1]
Major vault protein	Mvp	Promote HDM2-dependent loss of p53 for HCC development [2]
Flap endonuclease 1	Fen1	Promote HCC through enhanced USP7/MDM2-mediated P53 inactivation [3]
Peroxiredoxin-4	Prdx4	Promote tumorigenesis and metastasis via β-catenin pathway [4]
Ribose-5-phosphate isomerase	Rpia	Promote HCC via PP2A and ERK signaling [5]
Bromodomain-containing protein 2	Brd2	Promote HCC via Wnt/β-catenin pathway [6]
Mitochondrial fission 1 protein	Fis1	Promote autophagy and HCC cells survival via NFKB and TP53 pathway ROS regulation [7]
Fatty acid-binding protein 5	Fabp5	Promote tumor angiogenesis and activation of IL6 / STAT3 / VEGFA pathway in HCC [8]
Protein transport protein Sec61 subunit alpha isoform 1	Sec61a1	Promote cell proliferation and migration [9]
Proteasome activator subunit 4	Psme4	Promote the development of HCC via mTOR signaling pathway [10]
C-terminal-binding protein 1	Ctbp1	Play a key role in hypoxia-induced EMT and sarcomatoid transformation [11]<br>Promote cell proliferation in HCC by regulating miR-623/cell cycle protein D1 axis [12]

REFERENCES

1. Lou G, Dong X, Xia C, Ye B, Yan Q, Wu S, et al. Direct targeting sperm-associated antigen 9 by miR-141 influences hepatocellular carcinoma cell growth and metastasis via JNK pathway. J Exp Clin Cancer Res. 2016; 35: 14.
2. Yu H, Li M, He R, Fang P, Wang Q, Yi Y, et al. Major Vault Protein Promotes Hepatocellular Carcinoma Through Targeting Interferon Regulatory Factor 2 and Decreasing p53 Activity. Hepatology. 2020; 72: 518-34.
3. Bian S, Ni W, Zhu M, Zhang X, Qiang Y, Zhang J, et al. Flap endonuclease 1 Facilitated Hepatocellular Carcinoma Progression by Enhancing USP7/MDM2-mediated P53 Inactivation. Int J Biol Sci. 2022; 18: 1022-38.
4. Wang W, Shen XB, Huang DB, Jia W, Liu WB, He YF. Peroxiredoxin 4 suppresses anoikis and augments growth and metastasis of hepatocellular carcinoma cells through the beta-catenin/ID2 pathway. Cell Oncol (Dordr). 2019; 42: 769-81.
5. Ciou SC, Chou YT, Liu YL, Nieh YC, Lu JW, Huang SF, et al. Ribose-5-phosphate isomerase A regulates hepatocarcinogenesis via PP2A and ERK signaling. Int J Cancer. 2015; 137: 104-15.
6. Fang D, Wang MR, Guan JL, Han YY, Sheng JQ, Tian DA, et al. Bromodomain-containing protein 9 promotes hepatocellular carcinoma progression via activating the Wnt/beta-catenin signaling pathway. Exp Cell Res. 2021; 406: 112727.
7. Huang Q, Zhan L, Cao H, Li J, Lyu Y, Guo X, et al. Increased mitochondrial fission promotes autophagy and hepatocellular carcinoma cell survival through the ROS-modulated coordinated regulation of the NFKB and TP53 pathways. Autophagy. 2016; 12: 999-1014.
8. Pan L, Xiao H, Liao R, Chen Q, Peng C, Zhang Y, et al. Fatty acid binding protein 5 promotes tumor angiogenesis and activates the IL6/STAT3/VEGFA pathway in hepatocellular carcinoma. Biomed Pharmacother. 2018; 106: 68-76.
9. Fa X, Song P, Fu Y, Deng Y, Liu K. Long non-coding RNA VPS9D1-AS1 facilitates cell proliferation, migration and stemness in hepatocellular carcinoma. Cancer Cell Int. 2021; 21: 131.
10. Ge S, Huang H, Huang W, Ji R, Chen J, Wu S, et al. PSME4 Activates mTOR Signaling and Promotes the Malignant Progression of Hepatocellular Carcinoma. Int J Gen Med. 2022; 15: 885-95.
11. Zhang X, Wang X, Jia L, Yang Y, Yang F, Xiao S. CtBP1 Mediates Hypoxia-Induced Sarcomatoid Transformation in Hepatocellular Carcinoma. J Hepatocell Carcinoma. 2022; 9: 57-67.
12. Wang M, Zhao H. LncRNA CTBP1-AS2 Promotes Cell Proliferation in Hepatocellular Carcinoma by Regulating the miR-623/Cyclin D1 Axis. Cancer Biother Radiopharm. 2020; 35: 765-70.
